# A natural programmable metamaterial controls 3D curvature of compound eyes

**DOI:** 10.1038/s41467-026-74276-6

**Published:** 2026-06-30

**Authors:** Juan Garrido-García, Rhian F. Walther, Jesús Torres-Tirado, Jesús A. Andrés-San Román, José A. Sanz-Herrera, Franck Pichaud, Fernando Casares, Luis M. Escudero

**Affiliations:** 1 Departamento de Matemática Aplicada I, Universidad de Sevilla and Instituto de Biomedicina de Sevilla (IBiS), Hospital Universitario Virgen del Rocío/CSIC/Universidad de Sevilla, Seville, Spain; 2 Epithelial Biology Group, Laboratory for Molecular Cell Biology (LMCB), University College London, London, UK; 3 CABD, CSIC/Universidad Pablo de Olavide/Junta de Andalucía, Seville, Spain; 4 Departamento de Biología Celular, Facultad de Biología, Universidad de Sevilla and Instituto de Biomedicina de Sevilla (IBiS), Hospital Universitario Virgen del Rocío/CSIC/Universidad de Sevilla, Seville, Spain; 5 Escuela Técnica Superior de Ingeniería. Universidad de Sevilla, and Instituto de Biomedicina de Sevilla (IBiS), Hospital Universitario Virgen del Rocío/CSIC/Universidad de Sevilla, Seville, Spain; 6 Institute for the Physics of Living Systems, University College London, London, UK; 7 CIBERNED, Network Center for Biomedical Research in Neurodegenerative Diseases, National Institute of Health Carlos III, Madrid, Spain

**Keywords:** Morphogenesis, Biomaterials, Biophysics

## Abstract

Panoramic vision of the convex compound eyes, common to insects and crustaceans, relies on micrometer-scale curvature variations. These variations generate specialized visual zones adapted to specific tasks, including detecting prey, mates, or predators. However, how such fine-scale curvature is encoded during development remains unknown. We find in *Drosophila melanogaster* that the basal surface of the developing retina is organized as a supracellular triangular mesh where the size of these triangles is distributed in a species-specific 2D pattern. Functional experiments using genetic perturbations, together with computational modeling, support the notion that this pattern guides adult eye local curvature. A similar pattern in the *Drosophila mauritania* developing retina indicates an evolutionary conservation of this mechanism. Our findings identify a mechanism of morphogenesis where fine-scale 3D curvature is programmed in the 2D patterning of a tissue with metamaterial properties. This mechanism provides a framework for designing shape-programmable 3D biological surfaces, with broad implications from synthetic morphogenesis to clinical applications.

## Abstract (web summary)

This article uncovers a natural, programmable metamaterial in the developing Drosophila eye. The precise 3D curvature of the adult compound eye is programmed during development by a microscopic, 2D triangular mesh blueprint lining the base of the retina.

## Introduction

**Fig. 1:**
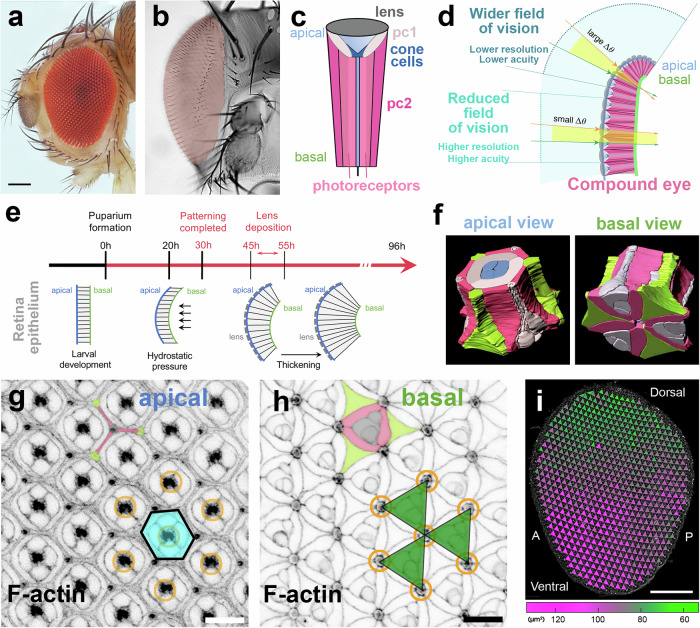
A supracellular triangular mesh patterns the basal pupal retina of *Drosophila.* **a**, **b** *D. melanogaster* compound eye. Lateral (**a**) and frontal (**b**) views (**b**, eye pseudocolored in light pink). **c** Schematic of an ommatidium with all major cell types labeled. **d** Eye cross-section illustrating how local curvature differences affect the field of vision and image resolution; ΔΦ is the interommatidial angle. **e** Developmental timeline of eye morphogenesis at 25 °C, and milestones; hAPF hours after pupa formation. **f** 3D reconstruction of an ommatidium. Apical (left) and basal (right) views. 1°PC (light pink), 2°PC (pink), 3°PC (yellow) and bristle cell complex (grey). PC pigment cell. Apical (**g**) and basal (**h**) confocal views through a 42 hAPF retina. Ommatidia form a hexagonal lattice apically (cyan hexagon). Orange rings mark the position of the grommet (**h**), the photoreceptors’ axon exit point, which in more apical sections aligns with the longitudinal axis of the ommatidium (**g**). Basally, elongated 2°PC profiles (pink) form a triangular mesh hinged at the grommets. The derived triangles (green) overlap the basal cell surfaces of the bristle cell complex (pseudocolored in grey), complementary to the 3°PC profiles (yellow). **i** View of a whole 42 hAPF retina with superimposed triangular mesh. Triangle size is color-coded (green-to-purple), revealing a dorsal/posterior to ventral/anterior gradient of increasing triangle size. A anterior, P posterior. Scale bars: **a**, **i** = 100 µm; **g**, **h** = 10 µm.

Morphogenesis, the controlled shaping of living materials, is essential for the correct organization and function of complex organs. A paradigmatic example of how form impacts function is the insect compound eye, an optical device of great precision. It consists of a crystalline packing of unit eyes, called ommatidia, into a convex, dome-like structure (Fig. 1a, b). Each ommatidium comprises a central photodetection cartridge capped by a facet lens and ensheathed by a layer of ancillary cells (Fig. 1c). Importantly, curvature can vary across the eye, and these small-scale curvature anisotropies (what we will call heretofore “local curvature”), which make the eye deviate from a uniform dome, often differ between species^1^. These variations reflect functional needs, as curvature variation modifies visual performance. Thus, zones of low local curvature focus many ommatidia onto a narrow region, resulting in high spatial-resolution vision, while zones with higher local curvature expand the field of view. The combination of local curvature and lens diameter, with larger lenses providing greater light sensitivity, gives compound eyes a range of optical properties, vital for supporting predation, mating, or escape responses^2,3^ (Fig. 1d). Therefore, there must be mechanisms responsible for controlling local, small-scale curvature variations during the development of the compound eyes, as this trait critically impacts their visual performance.

The development of the compound eye is best understood in the fruitfly *Drosophila melanogaster*^4–6^. Cell differentiation and patterning of the retina start in the late larval stage and continue after the larva begins its metamorphosis, in the early pupa. It is also during pupal life that the retina morphs into a 3D convex cup (Fig. 1e). This transformation likely starts with the building up of hydrostatic pressure within the pupa due to the contraction of the abdominal muscles. This pressure would provoke the eversion of the head, including the developing retina, and the acquisition of a general convex curvature at around 20 h after puparium formation (hAPF)^7–10^. Puncturing experiments performed by Lancaster and coworkers^10^ show that a reduction of internal pressure as early as 20 hAPF results in loss of retinal curvature, supporting this idea. Retinal curvature keeps increasing steadily until 45–50 hAPF. From this time point, the retina will further increase its curvature very little^10^. After metamorphosis, the convex adult *Drosophila* eye shows a non-homogeneous curvature, as expected for the eye having local visual specializations (Fig. 1a, b). How the small-scale variations of the eye curvature are encoded in the fabric of the retinal tissue is not known.

In this work, we show that a patterned triangular mesh at the basal surface of the developing fly retina guides the species-specific curvature of the adult eye. This 2D-to-3D translation mirrors structural engineering principles used to program the shape of a 3D curved object. The patterned triangular mesh we report here confers programmable metamaterial properties to the tissue, revealing a convergence between biological evolution and human design strategies.

## Results

### A patterned triangular mesh tiles the pupal retina

Early during pupal development, retinal cells acquire their position and remodel their morphology to shape the ommatidium as a prism (Fig. 1f and Supplementary Fig. 1;^11–13^). At this stage, apical confocal views of the retinal epithelium show the hexagonal lattice of ancillary interommatidial cells (IOCs) consisting of the secondary (2°PC) and tertiary pigment cells (3°PC), the four lens-secreting cone cells and the sensory bristle cells complex (Fig. 1g and Supplementary Fig. 1). Basal confocal views show the cellular profiles of the IOCs coordinating their attachment around the afferent photoreceptor axons. In this organization, the IOCs form supracellular rings, called “grommets,” rich in extracellular matrix (ECM), which act as portholes through which the photoreceptor axons exit the retina^11–13^. On this basal surface, the elongated feet of the 2°PCs form triangles with the grommets as vertices, creating a continuous triangular multicellular mesh that spans the entire tissue (Fig. 1h)^13^.

Mechanical metamaterials are designed structures that consist of repetitive connected units. They are called “meta” materials because their unique mechanical properties come from how the units work together, not just from the material they are made of refs. ^14–16^. Combining physics, engineering and computer science, it has been possible to design *programmable* metamaterials, where the rational distribution of the units in 2D controls the 3D shape as loading is applied to the metamaterial^14,17^. One type of programmable metamaterials are bidimensional meshes in which the unit elements are triangles. In these “2D-triangular meshes” local curvature can be programmed by varying the size of the triangles throughout the mesh^18–20^ (Box 1). The similarities between these metamaterials and the multicellular triangular mesh of the retina led us to hypothesize that the developing compound eye might behave like a natural metamaterial, where the mesh formed by the 2°PC would encode local curvature.

For this hypothesis to be true, three conditions must be met. First, the triangles of the mesh should be distributed across the 2D retina in a nonuniform, stereotyped pattern. Second, this 2D patterning should be instructive in generating species-specific fine-scale 3D eye local curvature. And third, perturbing the integrity of the triangular mesh should result in the eye losing its species-specific local curvature.

To analyze the pattern of triangle size of the basal mesh, we imaged and segmented the basal surface of pupal retinas at 44 h APF, a stage where this tissue surface has completed its patterning. Meeting our first condition, we found that the size of the triangles defined by the lattice of 2°PCs is distributed as a gradient of increasing size from dorsal/posterior to ventral/anterior across the retina (Fig. 1i, Supplementary Fig. 1 and “Methods”). Confocal imaging of live pupal retinas showed that this gradient was present in vivo (Supplementary Fig. 2). Therefore, the mesh of triangles is micropatterned in 2D.

Box 1 Generation and curvature quantification of 3D surfaces

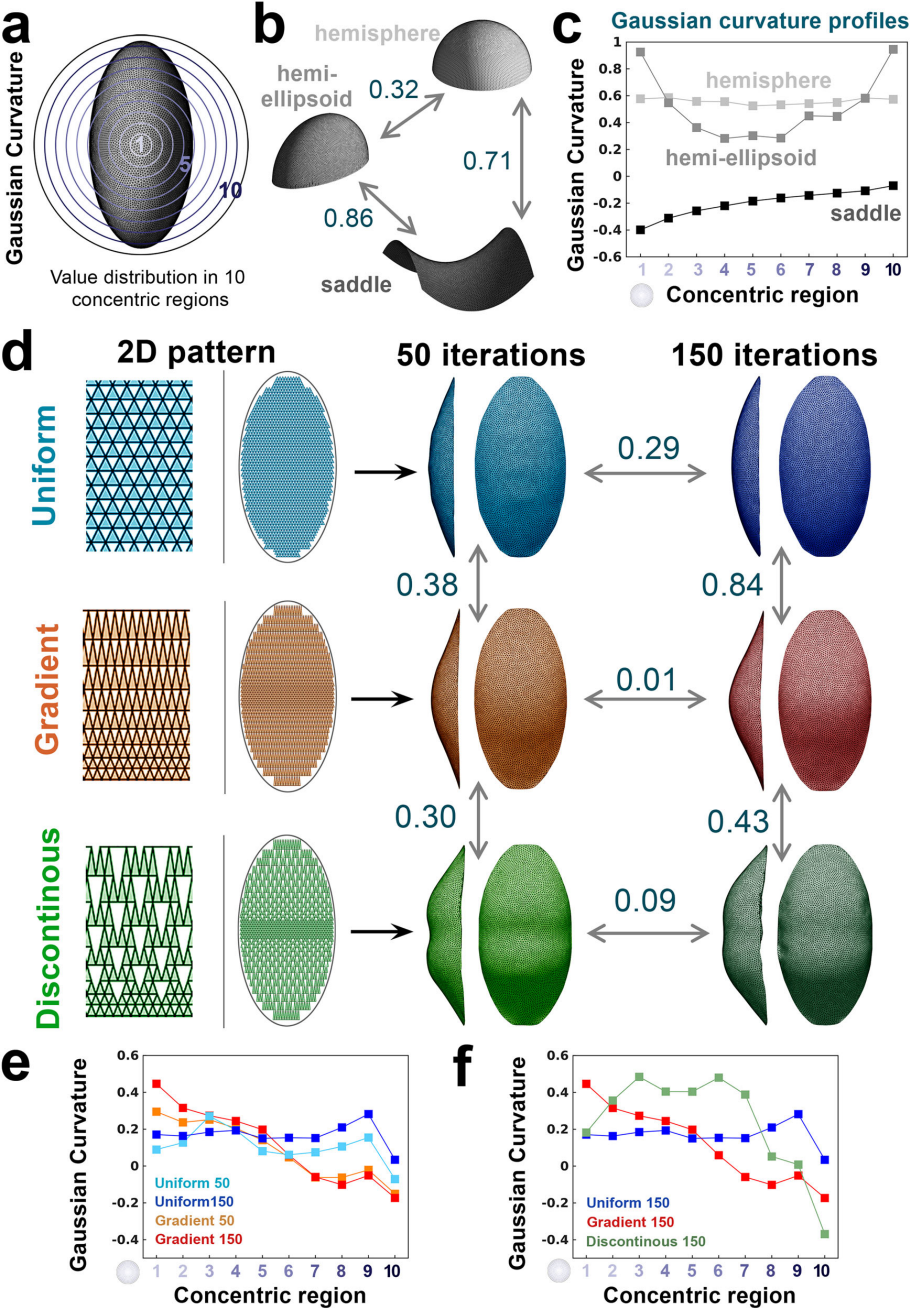

(**a**) To compare dome-like surfaces, such as the *Drosophila* eye, it is necessary to quantify the 3D curvature distribution of the entire surface. Therefore, we calculated a curvature metric, the “Gaussian curvature” metric, across the surfaces of interest. With this method, each curved surface is divided into ten concentric regions, and the integral of the dimensionless Gaussian curvature is calculated within each region (see “Methods” and Supplementary Methods). (**b**) As reference shapes, we use three idealized 3D surfaces: hemisphere, hemi-ellipsoid, and saddle. These illustrate typical curvature types: constant positive, varying positive, and negative curvature, respectively. The rigid perimeter is the same for all three patterns. (**c**) Each shape’s curvature is represented as a set of ten values (one per concentric region, as in a), generating a *Gaussian curvature profile* (see “Methods”). The Gaussian curvature profiles are compared in a pairwise manner using the Pearson correlation coefficient, thereby defining the Gaussian curvature metric, which quantifies the similarity of curvature distributions between the three reference shapes shown in (**b**). The hemisphere and hemi-ellipsoid are similar (lower value), while the saddle is distinctly different from both. (**d**) To model curved metamaterial-like tissue formation, pressure is applied on a 2D triangular mesh enclosed in an elliptical rigid frame. We illustrate the differences in curvature distribution attained using three patterns of triangle size: uniform: identical triangle sizes (blue); gradient: triangle sizes increase linearly from the equator toward the poles (orange); discontinuous: same as the gradient but homogeneously removing 20% of the triangles in the upper bottom regions. Each 2D mesh is inflated over a series of computational iterations, and the resulting 3D shape is then processed for curvature analysis (Supplementary Fig. 4). The panel shows side views (yz and xz) of the processed surfaces generated by each pattern, at 50 (lighter colors) and 150 (darker colors) iterations. The xz views highlight the overall shape and fine irregularities. The numbers indicate the Gaussian metric values obtained from comparisons between the surfaces linked by the double-headed arrows. The smaller the value, the more similar the shapes. (**e**) The Gaussian curvature profiles of the uniform (cyan/blue) and gradient (orange/red) surfaces are plotted after 50 and 150 iterations. The gradient pattern yields very similar 3D Gaussian curvature profiles at both steps (Gaussian metric <0.1). In contrast, the uniform pattern develops more noticeable differences in Gaussian curvature profiles between 50 and 150 steps (Gaussian metric = 0.29), reflecting emerging irregularities during inflation. (**f**) Final Gaussian curvature profiles after 150 iterations for the three patterns show poor correlation between them. Their pairwise Gaussian metric values are indicated in (**d**), reflecting the divergence of their 3D curvatures.

### The patterned triangular mesh encodes the local curvature of the eye

**Fig. 2:**
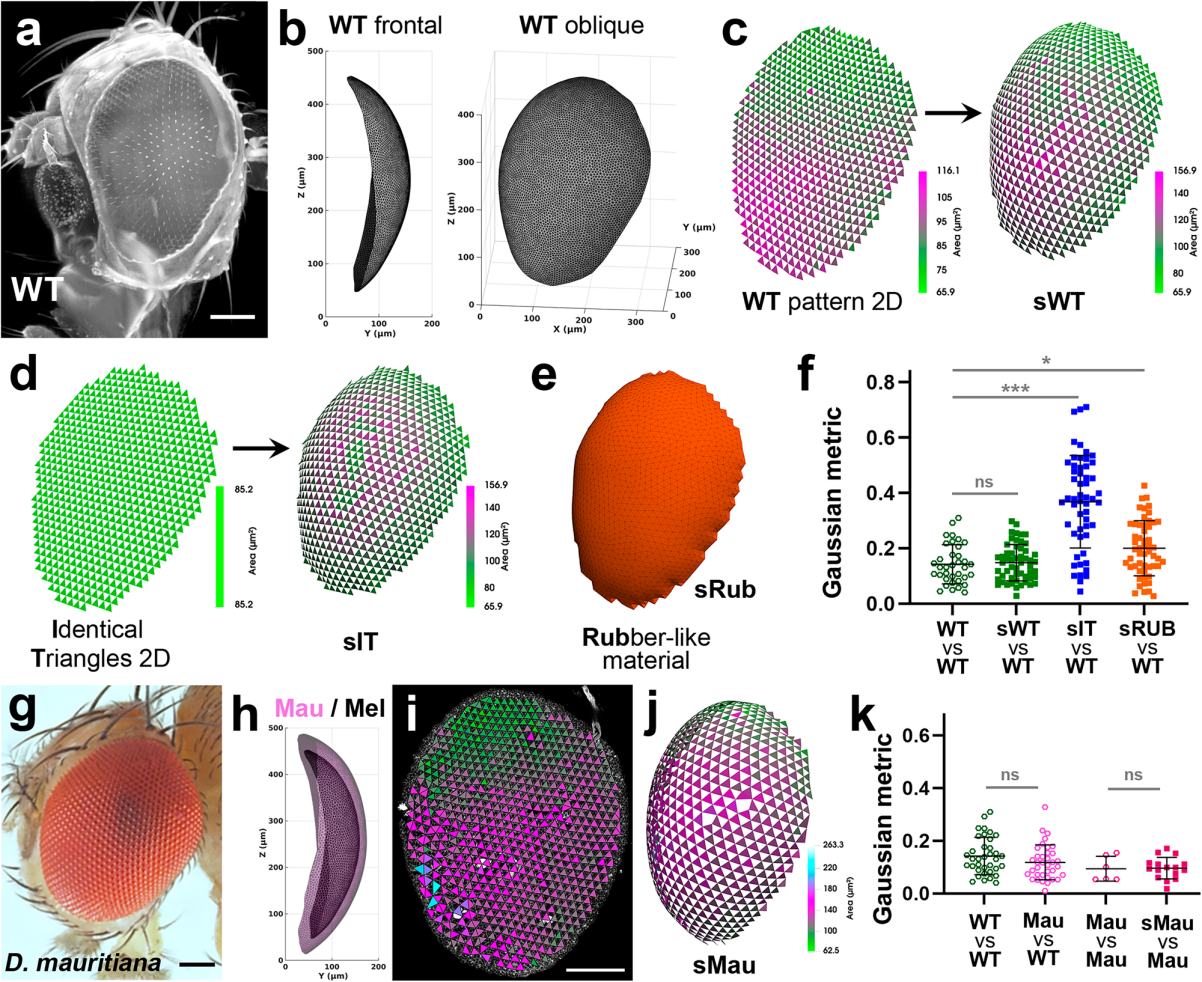
A physical model reproduces retinal curvature across *Drosophila* species. **a** Lateral view of a wild-type (WT) *D. melanogaster* head imaged using light-sheet microscopy. **b** Segmented eye surface (WT, frontal and oblique views) extracted from (**a**). **c** A patterned 2D triangle mesh extracted from (**a**) *D. melanogaster* pupal retina is used to generate a simulated 3D surface (sWT; see “Methods”). **d** For comparison, a 2D mesh consisting of triangles uniform in size, generates the sIT surface. **e** A third simulation (sRub) mimics a rubber-like material with the same perimeter. **f** Statistical analysis of the sets of Gaussian metric values for the comparisons of the resulting 3D surfaces (see Box 1); each dot represents one pair-wise comparison among 9 WT and 6 sWT, sIT and sRUB (ns, *p* > 0.05 (>0.99), *p < 0.05 (0.04); ***p < 0.001 (<0.001), Kruskal–Wallis). sWT is the only simulation that reproduces WT curvature distribution. **g** Lateral view of an adult *D. mauritiana* head. **h** Frontal view of segmented *D. mauritiana* (Mau, pink) and *D. melanogaster* (Mel, grey) eyes, superimposed for comparison. **i** The *D. mauritiana* pupal retina displays a triangle s**i**ze gradient similar to *D. melanogaster*. **j** sMau: the simulated 3D surface based on the *D. mauritiana* triangle pattern. **k** *D. melanogaster* (WT) and *D. mauritiana* (Mau) eyes are similar in curvature distribution, and sMau accurately reproduces the curvature of the adult *D. mauritiana* eye, showing a strong match with empirical data. Each dot represents one pair-wise comparison among 9 WT, 4 Mau and 4 sMau (ns, *p* > 0.05, Mann–Whitney, ordinary one-way Anova). Scale bars: **a**, **g**, **i** = 100 µm. Triangle size colored according to scale in (**c**, **d**, **i**, **j**). Data shown as mean ± s.d.

Next, to test whether the 2D triangular mesh encodes the local curvature of the adult eye, we developed a physical model of this mesh. The model was implemented as a finite element method (FEM)^21^ to simulate the mechanical response of thin mesh structures using triangles as mesh units. This model is capable of capturing both membrane behaviors, responsible for in-plane area changes, and bending behaviors, responsible for out-of-plane curved deformations. Boundary conditions are imposed on the vertices at the perimeter by fixing all their degrees of freedom to zero, implying that these vertices cannot undergo any displacement or rotation (see Supplementary Methods). On the remaining vertices, a pressure load is applied along the normal direction, computed via an area-weighted averaging algorithm (see Supplementary Methods). Using this model, we could program any distribution of triangle size within a given perimeter and simulate the resultant 3D curvature upon applying pressure to the mesh (Box 1 and Supplementary Methods). To analyze and compare curvature between samples, we used a Gaussian curvature-based metric (Box 1, Supplementary Data 1 and “Methods”). Finally, as our goal was to compare computational and biological structures, we developed a computational pipeline to segment images of adult eyes, enabling precise measurement of the curvature distribution that captures the local curvature of the eye surface (Fig. 2a, b, Supplementary Figs. 3, 4, Supplementary Data 2, Supplementary Video 1 and “Methods”).

To explore the link between triangular mesh and curvature, we programed three types of 2D triangle mesh: (i) wild-type *Drosophila melanogaster* meshes computationally segmented from 42 hAPF retinas, “sWT”; (ii) uniform meshes consisting of identical triangles, “sIT”; and (iii) continuous, rubber-like fine meshes consisting of smaller triangles with random orientations, “sRub” (see “Methods” for a detailed description; Fig. 2c–e, Supplementary Figs. 3 and 4). sRub was included to mimic the behavior of a homogeneous, non-programmed material^22^. To simulate the adult *Drosophila* eyes, we deployed each mesh within the perimeters measured from pupal retinas, inflating each mesh until they best matched the mean depth of the wild-type adult eyes (Fig. 2b–e, Supplementary Figs. 3, 4, Supplementary Video 2, “Methods,” Supplementary Data 2 and 3). Finally, in order to estimate quantitatively how similar the 3D curvature distributions of two surfaces are, we computed the Gaussian metric in a pairwise manner (Supplementary Data 4). We found that local curvature of WT eyes was comparable across our samples (low values of Gaussian metric of “WT vs. WT” in Fig. 2f, Supplementary Figs. 1,3, Supplementary Data 5,and “Methods”). Remarkably, and validating our computational model, our analysis showed the curvature distribution of the simulated WT (sWT) was very similar to the one we measured on WT adult retinas. In good agreement with this, curvature distributions corresponding to sIT or sRub were significantly different (Fig. 2f, Supplementary Data 5, and “Methods”). Therefore, only the meshes consisting of size distributions of triangles derived from WT retinas were able to morph into the adult 3D eye curvature distribution we measured from adult eyes.

To further challenge our hypothesis, we tested whether there was a correlation between eye curvature distribution and the pattern of triangles in the retinal mesh in other fly species. For this, we chose to examine the eye of *Drosophila mauritiana*, a species that diverged from *D. melanogaster* about 4 Myrs ago^23^ (Fig. 2g–k and Supplementary Fig. 5). *D. mauritiana* has larger eyes (Fig. 2g and Supplementary Fig. 5) as its retina comprises more and larger ommatidia when compared to *D. melanogaster*^24^. Comparing *D. mauritiana* (“Mau”) and *D. melanogaster* (“WT”, “Mel” in Fig. 2h) eyes showed that, despite their size difference, they have a very similar curvature distribution (Fig. 2k, Supplementary Data 2 and 5). According to our hypothesis, the retina of *D. mauritiana* should present a patterned triangular mesh similar to that of *D. melanogaster*. Examining the developing retinal basal surface of these animals revealed this is indeed the case (Fig. 2i; compare with Fig. 1i). Moreover, incorporating the segmented triangular mesh into our computational model predicted the fine-scale local curvature of the *D. mauritiana* eye with great accuracy (Fig. 2k, see also Supplementary Fig. 5 and Supplementary Data 5). These results further indicate that during retinal development, the 2D patterned triangular mesh encodes the local curvature of the eye at least across the about 4 MYrs separating *D. melanogaster* and *D. mauritania*^23^.

### Disruption of the triangular mesh alters the curvature of the compound eye

**Fig. 3:**
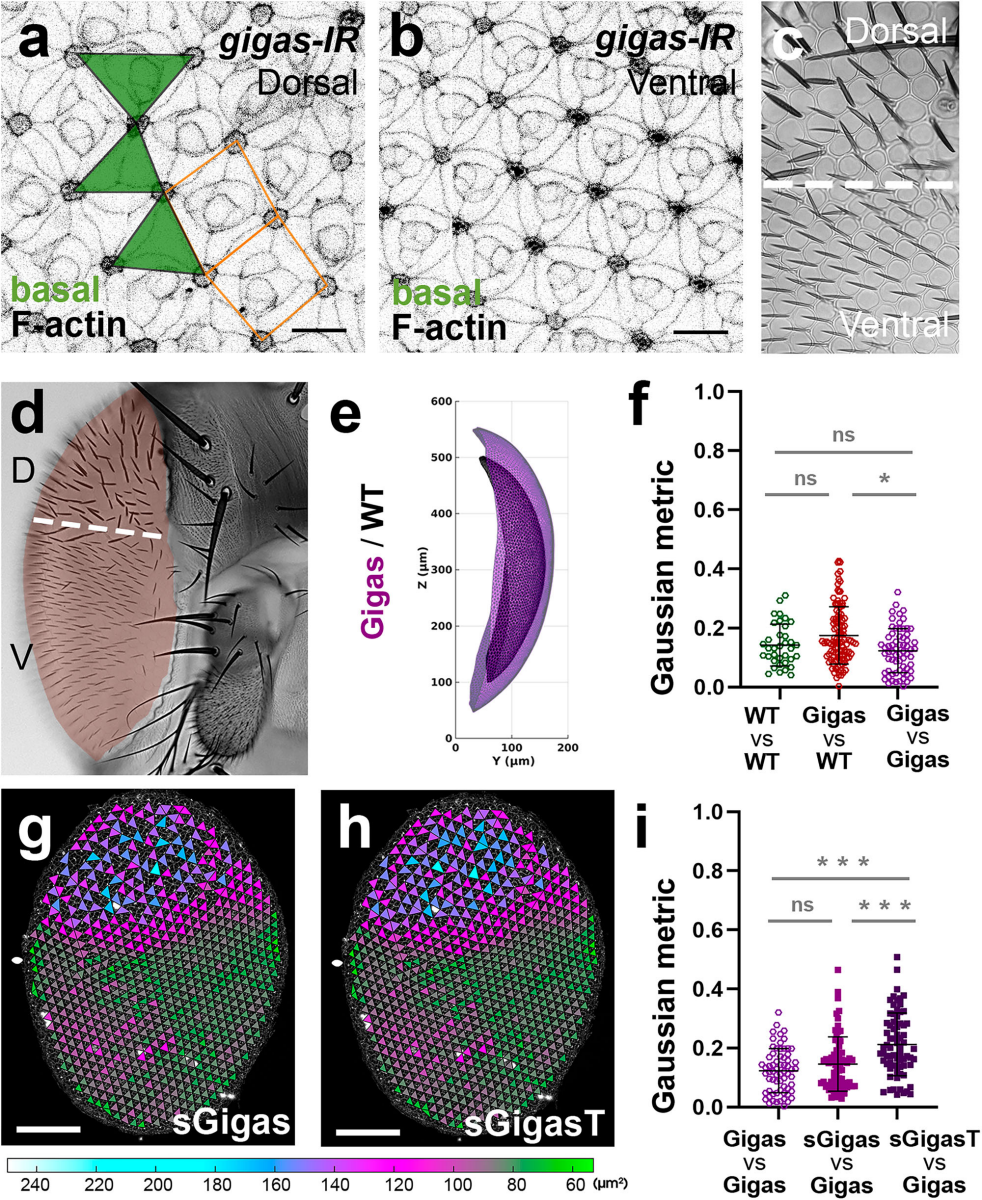
Alteration of the triangle pattern in a region. Confocal views through a *iro-G4*>*gigas-IR* pupal retina stained with phalloidin to visualize the F-actin at the basal surface of the retina in the dorsal (**a**) and ventral regions (**b**). **c** Close-up view of the surface of a *gigas-IR* adult eye, showing the size difference between dorsal and ventral lenses. The dashed line marks the approximate limit between the perturbed (dorsal) and unperturbed (ventral) ommatidia. **d** Frontal views of a *gigas-IR* adult eye, showing the alteration of the dorsal part of the eye. **e** Frontal view of segmented *gigas-IR* (gigas, purple) and WT (grey) eyes, superimposed for comparison. **f** Comparison of curvature. The Gaussian metric of gigas and WT does not show significant differences. However, the curvature of the Gigas eyes differs if compared with themselves or with WT eyes. Each dot represents one pair-wise comparison among 9 WT and 12 gigas (ns, *p* > 0.05, *p < 0.05, Kruskal–Wallis, one-sided). **g** Pattern of triangles in a gigas mutant pupa after eliminating 25% of the triangles on the dorsal region. **h** Pattern of triangles in a gigas mutant pupa, considering all grommets as vertices. **i** Comparison of curvature. The el**i**mination of the 25% of triangles enables sGigas eyes to adopt a similar curvature to aGigas. Each dot represents one pair-wise comparison among 12 gigas and 6 sGigas and sGigasT (ns, *p* > 0.05 (0.59), ***p < 0.001 (<0.001), Kruskal–Wallis, one-sided). Data shown as mean ± s.d.

In our hypothesis, alteration of the 2D triangular mesh lining the basal surface of the retina should affect the local curvature of the eye. To examine this, we sought to alter the pattern of triangle size across the retina. To this end, we drove an RNAi against *gigas (gigas-IR)*, a negative regulator of the insulin/mTOR pathway. Attenuation of *gigas* has been shown to increase cell size^25,26^. We reasoned that in *gigas-IR* retinas, the size of the triangles should be larger as well. To alter the triangle size pattern, the *gigas-IR* was specifically expressed in the dorsal third of the retina (“*gigas-IR*”, genotype *iro-Gal4>gigas-IR*, see “Methods”). As expected, the corresponding pupal retinas presented larger triangles in the dorsal region of the retina (Fig. 3a, b), as well as larger ommatidia (Fig. 3c). Globally, the adult *gigas-IR* eyes (“gigas”) were larger than those of controls (“WT”) (Fig. 3d, e). The statistical comparison between the distributions of Gaussian metrics detected subtle alterations of curvature, reflected by the statistically significant difference in the Gaussian metric values set obtained from gigas eyes (“gigas vs. gigas”) and from the comparison of gigas and WT eyes (“gigas vs. WT”) (Fig. 3f). We noted that in *gigas-IR* retinas not only the triangles were larger, but some of them were missing due to fusions among ommatidia (Fig. 3a). This amounted to about 25% of the triangles that should be connecting grommets. To simulate this situation, we built the mesh of triangles using the grommets as vertices, eliminating the triangles in the unpatterned areas (an average of 25% of the triangles of the affected region) (Fig. 3g and see “Methods”). Using this mesh (sGigas), our model generated curvature distribution similar to those we measured experimentally from *gigas-IR* adult eyes (Fig. 3i). However, if instead, we used a mesh considering all possible triangles (i.e., connecting all grommets as if there were 2°PC forming triangles in all cases (sGigasT)), the simulated surfaces failed to reproduce the curvature distribution of *gigas-IR* adult eyes (Fig. 3h, i). This fact not only indicates the high sensitivity of the curvature distribution to small changes in the mesh, but also that the basal triangular mesh results in predictable changes in the eye’s local curvature. Globally, all these results support the notion that the pattern of triangle sizes is, at least, a major guiding factor of the fine-scale local curvature of the eye.

**Fig. 4:**
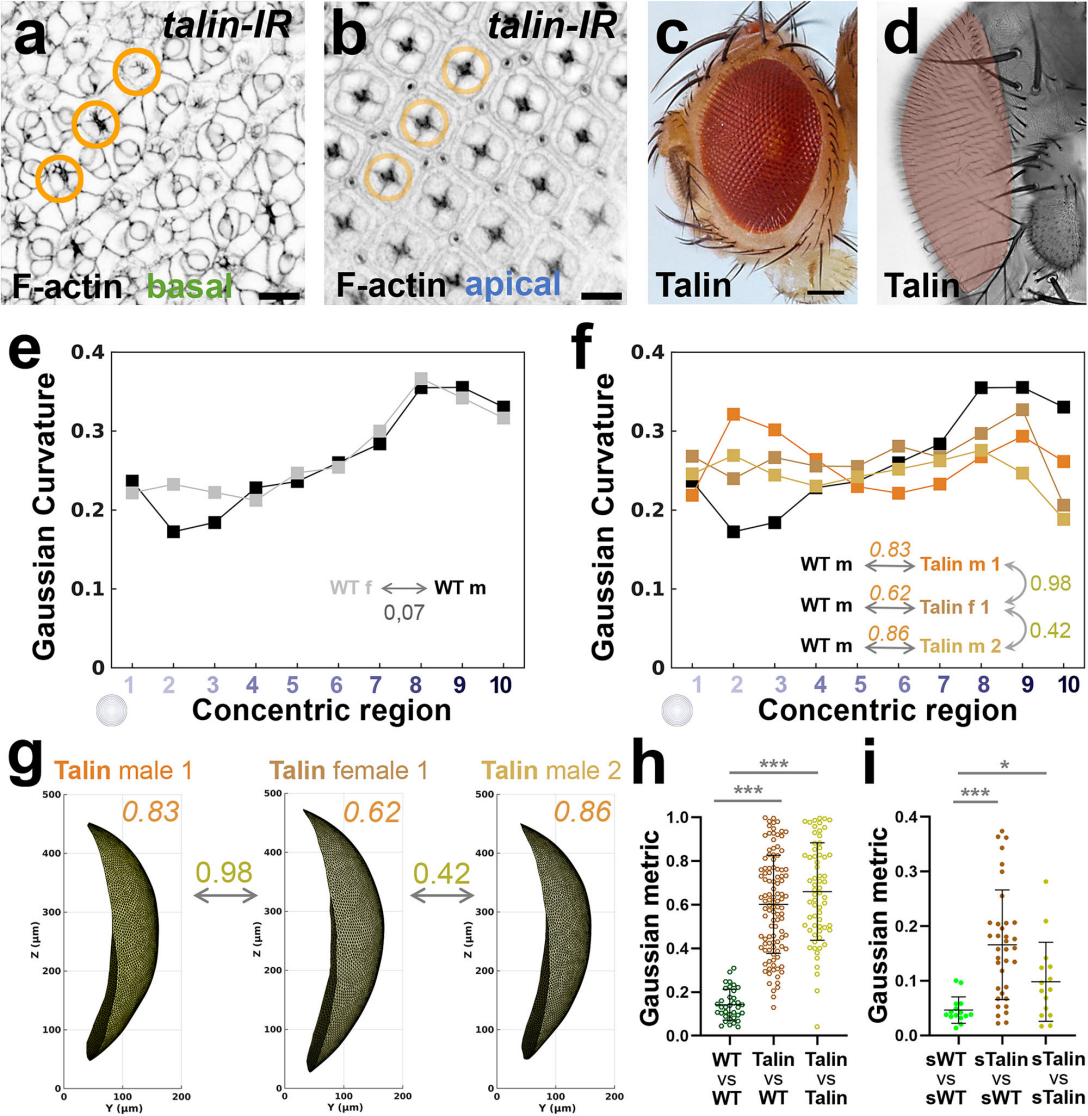
The patterned triangular mesh encodes for local curvature. Confocal views through a *talin-IR* pupal retina stained with phalloidin to visualize the F-actin at the basal (**a**) and apical (**b**) surface of the retina. Orange ring marks the ommatidial central axis. Lateral (**c**) and frontal (**d**) views of a *talin-IR* adult eye (“talin” in the figure), showing that these eyes are curved and present minor alterations. **e** Reference Gaussian curvature profiles for a WT eye from a female (grey) and a male (black). Their similarity is indicated by a very low value of their pairwise Gaussian metric (0.07). **f** Combined dataset of Gaussian curvature profiles, including a WT and three talin surfaces, was used to compute the pairwise Gaussian metric. The Gaussian curvature profiles of talin eyes are consistently distinct from WT. **g** 3D reconstructions of three segmented talin adult eyes. Bronze, italicized numbers indicate the Gaussian metric calculated from pairwise comparisons between each talin surface and a wild-type (WT) male retina (see **f**). Gold numbers show the metric between each pair of talin surfaces. **h** Statistical analysis of the sets of Gaussian metric values for these comparisons, showing that talin eyes differ significantly from WT and display higher variability. Each dot represents one pair-wise comparison among 9 WT and 12 talin (***p < 0.001, Kruskal–Wallis). **i** Equivalent comparisons using computationally generated reference surfaces confirm these trends. Each dot represents one pair-wise comparison among 6 sWT and 6 sTalin (***p < 0.001, *p < 0.05, Welch’s test). Scale bars: **a**, **b** = 10 µm; **c** = 100 µm. Data shown as mean ± s.d.

Next, to further examine the relationship between basal triangle mesh and curvature distribution, we genetically disrupted the basal mesh. To do this, we drove an RNAi to target the expression of *talin* (*talin-IR*), a protein required for Integrin-mediated attachment of the 2°PCs to the grommet^12,13,27^ (“*talin-IR*”, genotype *GMR-Gal4>talin-IR*, see “Methods”). *GMR-Gal4* is expressed in all retinal cells. In *talin-IR* retinas, the detachment of basal cells from the extracellular matrix causes the disconnection of the triangle mesh (Fig. 4a and Supplementary Fig. 6). The quantification of the hexagonal pattern on the apical surface of these retinas revealed the apical patterning remained largely unaffected (Fig. 4b, Supplementary Fig. 6, and refs. ^12,13,28^). In our model, we expect this should lead to a loss of metamaterial properties of the retina (i.e., the curvature distribution of the adult eye should resemble that of a homogeneous material, like rubber, losing its stereotypic fine-scale local curvature). While the *talin-IR* eyes were still dome-shaped (Fig. 4c, d, adult eye labelled as “talin” in the figure), their curvature distribution was markedly different from that of WT eyes (Fig. 4e–h and Supplementary Data 5). Notably, our curvature measurements showed disruption of the basal mesh led to a large curvature distribution variability (Fig. 4h and Supplementary Data 5). From this, we conclude that the basal triangular mesh is not only needed for programming the local curvature of the adult eyes, but also is required for its robustness. To investigate this further, we simulated 3D surfaces using the rubber material, described in Fig. 2e, which we framed within the perimeters of *talin-IR* retinas (“sTalin”, Supplementary Fig. 6 and “Methods”). This simulation led to an increase in curvature distribution variability similar to the one we measured for adult talin eyes (Fig. 4i and Supplementary Data 5). We obtained equivalent results when degrading the basal extracellular matrix (ECM) of the retina by expressing the metalloproteinase Mmp2^29^ (*“Mmp2*”, genotype *GMR-Gal4*>*UAS-Mmp2*, see “Methods”). In these retinas, cells lose their basal connections to the ECM, leading to a complete loss of the triangular mesh, as in *talin-IR* retinas (Supplementary Fig. 7). As it was the case with talin, Mmp2 eyes showed an altered curvature distribution relative to WT, which closely resembles that obtained when simulating curvature of a rubber-like material (Supplementary Fig. 7 and “Methods”). Altogether, our results identify the basal multicellular triangular mesh of the pupal retina, with its pattern of triangle sizes, as a major mechanism encoding the fine-scale local curvature variations of the compound eye and its robustness.

## Discussion

Since D’Arcy Thompson’s foundational work, it has been clear that understanding biological form requires examining the interplay between the principal forces and material properties at work during development^30^. During animal development, there are multiple examples where the physical forces that drive morphogenesis are spatiotemporally controlled at supracellular scales^31,32^. This idea is being pursued to engineer shapes in artificial tissues in vitro, through the programing of force asymmetries or microfabrication of 2D and 3D environments^33,34^. Here, we have found that the compound eye uses a similar strategy to program a 3D shape in vivo: the non-homogeneous multicellular pattern of the developing retina.

Our work indicates that the final local curvature of the adult eye, including its local variations, is acquired in two steps. The most likely force driving retinal inflation is hydrostatic pressure^7–9^. During the early stages of inflation, the retinal epithelium has not yet established its basal triangular mesh, and its curvature would resemble that of a uniform elastic material. However, midway through pupation, the 2°PCs interconnect through basal integrin-mediated contacts to establish a patterned triangular mesh^12,13^. From this point onward, the retina behaves as a metamaterial. This metamaterial quality emerges from the specific mechanical coupling between 2°PCs, which makes the mesh integral to the entire epithelium^35^. Interestingly, though, since the retina is a biological material, its metamaterial properties could be rendered nonuniform through genetic programming. In other words, the genetic program that drives retinal development must also control the spatial pattern of triangle sizes across the mesh. Consequently, during the second half of pupal life, and under the constant action of internal hydrostatic pressure, the curvature of the retina is locally modulated according to this pattern of triangle sizes. It follows that local modulations of the eye’s curvature can evolve through genetic changes that alter this spatial size pattern, as we demonstrate here by perturbing the dorsal region of the mesh. A key question moving forward is which genetic mechanisms translate positional information into cell size regulation across the tissue during normal development.

Part of the support for this mechanism comes from a new computational model capable of predicting the curvature distribution of the compound eye with great precision by using the patterned basal mesh of triangles we extracted from developing pupal retinas. The model captures these properties as follows. The material making the triangles is modeled as isotropic (i.e., with equal properties in all directions) and linearly elastic according to thin shell theory (Supplementary Data 3). The *t/h* ratio—with *t* being the thickness of the triangle sides, and *h* the triangle height—plays a fundamental role in the model, as it governs the relative stiffness of each of the triangles making up the mesh. Thus, variation in triangle area across the mesh allows this ratio to distribute mechanical forces differently according to the size pattern, such that curvature anisotropies emerge (as in “sWT”). In contrast, in the rubber-like material (“sRub”), the *t/h* ratio is constant throughout the mesh, precluding local curvature modulation. Although the model assumes uniform material properties, it is still possible that these properties vary across its basal surface in vivo due, for example, to local changes in the ECM composition or biophysical properties, as well as to variations in contractile properties of the basal actomyosin cytoskeleton^35,36^. Future experiments to physically measure the mechanical properties of the tissue will be necessary to challenge the structural assumptions of our simulations. However, the fact that our model is able to recreate the subtle local curvature of different compound eyes based solely on the pattern of triangle sizes suggests that this pattern is the dominant cause of the local curvature modulations.

Recent work in the *Drosophila* wing has shown that it is the heterogeneity of cell behaviors (including changes in the cell’s apical surface and cell neighbors) in the developing 2D wing epithelium that programs the 3D shape^37^. This contrasts with the mechanism we describe here for the eye, where a stereotypical supracellular pattern provides the basis for the fine-scale control of 3D curvature. In any case, these two scenarios illustrate that different organs may use distinct mechanisms for precise control of their shape, even within a single species.

To our knowledge, the developing retina of flies is the first instance of a natural metamaterial in which its properties are programmed genetically. The use of synthetic metamaterials with rationally designed properties is fast expanding^38^, with new applications, such as patches to give structural support to infarcted myocardium, vascular stents or wound dressings to aid skin healing^14^. By showing how local geometry can be embedded in tissue architecture, our work reveals a strategy allowing for the rational design of shape-programmable 3D biological surfaces, with potential implications extending from synthetic morphogenesis to clinical applications.

In addition to specifying the target morphology, a problem biological systems face is that of precision—reaching the species-specific morphology despite intrinsic and extrinsic noise. The need for precise curvature control has been made especially evident in studies of the *Drosophila* eye, where even subtle morphological defects can compromise optical function^39,40^. Moreover, local curvature variation must occur at the microscale, within a tissue only a few hundred microns across. Waddington proposed that phenotypic robustness should be the result of control mechanisms operating during development^41^. The phenomenon we describe here represents such a mechanism, where the robust and precise control derives from the metamaterial properties of the retina. The basal mesh seems to be better suited to encode retinal shape than the apical side. On the one hand, the basal surface is directly exposed to the pupal hydrostatic pressure. On the other hand, during pupal development, stress fibers build up along the elongated basal side of the secondary pigment cells, which form the triangle sides, which are known to mechanically integrate the retinal epithelium^35,36^. We also observed that the gradient in basal triangle size, from posterior/dorsal to anterior/ventral, mirrors a corresponding gradient in increasing lens size described in several *Drosophila* species, including *D. mauritiana* and *D. simulans*^42^. Since both local curvature and lens diameter influence visual acuity, it is plausible that control of ommatidial cell size co-regulates these two traits simultaneously. We have shown that *D. mauritania* shares with *D. melanogaster* the patterned basal triangular mesh. This fact suggests an evolutionary conservation of local curvature control. These two are related species separated by a relatively short evolutionary span. However, the organization of the compound eye is extremely conserved evolutionarily, not only within insects, but also in crustaceans, which together are grouped under Tetraconata (denoting their shared ommatidial structure, which includes four cone cells)^43–45^. Therefore, we envisage that the mechanism for local curvature control we have described in related *Drosophila* species may be conserved well beyond flies, something that deserves further investigation. Considering the long evolutionary history of the compound eye, dating back to the Cambrian and coinciding with the explosive diversification of arthropods (reviewed in ref. ^45^), it is tempting to speculate that mechanisms of curvature control and visual optimization, such as the one described here, might have played a role in the evolutionary success of insects and crustaceans.

## Methods

### Fly strains and genetics

Flies were raised on standard food at 25 °C. The following fly strains were used: *hth::YFP* (Kyoto:115109)^46^, *GMR-Gal4*^47^ (FlyBase: FBgn0020433; S0092-8674(00)81385-9 [pii]), *UAS-talin RNAi* (BDSC:33913)^48^, *D. mauritania* Tam-16 (gift from Alistair McGregor, Durham University, UK)^42^, *UAS-Mmp2* (BDSC:58704)^49^*, iro-Gal4*^50^; *UAS-gigas-RNAi* (P(KK100646)VIE-260B. Flybase: FBst0475275), ECad::GFP^51^.

### Antibody staining and imaging

Retinas of appropriately staged animals were dissected in PBS on ice and fixed in 4% paraformaldehyde for 20 min at room temperature (RT). Retinas were washed in PBS-Triton 0.3% (PBS-T), then stained with primary antibody in PBS-T for 2 h at RT or overnight at 4 °C. Retinas were washed in PBS-T and then stained with secondary antibodies for 2 h at RT or overnight at 4 °C. Retinas were mounted in Vectashield (Vectorlabs). The following primary antibodies were used: mouse N2 7A1 anti-Armadillo (1:200), mouse EXD B11M anti-Extradenticle (1:5) and rat DCAD2 anti-ECadherin (1:50). N2 7A1 Armadillo was deposited to the DSHB by Wieschaus, E. (DSHB Hybridoma Product N2 7A1 Armadillo)^52^. White, R. (DSHB Hybridoma Product EXD B11M), deposited EXD B11M to the DSHB^53^. DCAD2 was deposited to the DSHB by Uemura, T. (DSHB Hybridoma Product DCAD2)^54^. Anti-mouse or anti-rat secondary antibodies conjugated to CF 405S (Biotium, 20830) were used at 1:200 as appropriate, and ATTO 565 phalloidin (Sigma, 94072) was used at 0.4 µM to visualize F-actin. Images of fixed retinas were acquired on a Zeiss 900 confocal microscope using the tile scan function. For live imaging, *Ecad::GFP;* pupae^51^ were staged at 25 °C for 42 h. The pupal case was removed to expose the retina, and pupae were mounted as previously described in ref. ^55^. A Zeiss 900 confocal microscope was used to first image the dorsal region of the retina, then the pupae were repositioned to image the ventral region.

### Preparation of adult heads and light sheet confocal imaging

This protocol,  is based on Susaki^56^. All incubations were performed at RT with agitation.

*Dissection*: flies were euthanized in CO_2_ or on ice. They were then decapitated, and the heads were placed in a well containing 1× PBS. The proboscides were removed to allow further diffusion between the external medium and the interior of the head capsules. *Fixation*: the specimens were fixed in 4% paraformaldehyde in ethanol for 3–4 h and then washed three times in pure ethanol for 1 h each time. *Bleaching*: the heads were placed in tubes containing 10% H_2_O_2_ in ethanol until they were completely bleached (the time is variable and depends, for example, on whether the proboscis was fully removed or not). (Caution: this reaction produces oxygen. Leave the tube or well open during the first few hours of this step to allow the oxygen to escape. When the bubbling stops, the lid can be closed). This step may take from several days to 1, 5 weeks for adult heads. Change the medium if it becomes pigmented. After completion of bleaching, wash 3 times with PBS 1 × 1 h. *Clearing*: heads were incubated in 50% cubic-1/H_2_O for at least 3–6 h to overnight. Then, incubated in cubic-1 for 2 days. Next, incubated in cubic-2/PBS for at least 3–6 h to OV and then incubated in cubic-2 for 2 days. Finally, they were incubated 3 times in glycerol/PBS 50%: first for 3–4 h, second for OV, and third for 3–4 h.

Microscopy was performed with a Zeiss Lightsheet 7 under a 5× objective. The heads were mounted on 1:1 glycerol/PBS columns with 1% w/v low-melting agarose. The microscope chamber was filled with approximately 30 ml of 1:1 glycerol/PBS with a refractive index (RI) of 1.41. The software used was Zen Black (imaging) and Zen Blue (manual 3D reconstructions); the laser, 488 nm, which allows imaging of the cuticle autofluorescence.

### Statistical comparisons and interpretation

To evaluate the degree of similarity between simulations and adults (e.g., sWT and WT), we analyzed the statistical differences between the Gaussian metric values set obtained from WT vs. WT and sWT vs. WT. In this example, both types of samples presented low values of the Gaussian metric, so the absence of a statistically significant difference was interpreted as indicating similar curvature distribution between the two types of samples. We applied the same approach to compare the local curvature of WT and *talin-IR* samples. In this case, the Gaussian metric values set of WT vs. WT and talin vs. WT were significantly different, with the values for talin being substantially larger. This result was interpreted as evidence of a difference in local curvature and loss of robustness among the talin samples.

The comparative analysis of the different Gaussian curvature profiles vector was conducted using a univariate statistical framework (Supplementary Data 5 and 6). This approach enables the assessment of whether two datasets originate from populations with similar distributional properties. The protocol consisted of the following steps:

1. *Assessment of normality and homogeneity of variance*: for each pairwise comparison of Gaussian curvature distributions across genotypes, we first tested for normality using the Shapiro–Wilk test, and for homogeneity of variance using the two-sample F-test.
2. *Parametric testing with equal variances*: if both normality and equal variances were confirmed, we applied an unpaired t-test to assess differences in the means between the two groups. In case of multiple comparisons having one distribution as control, we applied a parametric ordinary one-way ANOVA test.
3. *Parametric testing with unequal variances*: in cases where the data were normally distributed but variances differed significantly, the two-tailed Welch’s t-test was employed as a more robust alternative. In case of multiple comparisons having one distribution as control, we applied a parametric ordinary one-way ANOVA test.
4. *Nonparametric testing*: when the assumption of normality was violated, we utilized the Wilcoxon rank-sum test (also known as the Mann–Whitney *U* test) to compare the medians of the two groups. In case of multiple comparisons having one distribution as control, we applied a Kruskal–Wallis test.

### Segmentation of confocal imaging data from 42 hAPF retinas and generation of the triangular mesh

Confocal stacks stained with ATTO 565 phalloidin (F-actin) were used to segment grommets without prior knowledge of retina orientation. Segmentation began with MATLAB’s *Volume Segmenter* (R2021b), followed by a deep learning pipeline based on a 3D U-Net CNN trained on a manually segmented stack^57^. The resulting probability maps were binarized with custom MATLAB code and manually corrected. Samples requiring excessive manual editing were excluded. Grommet centroids were then extracted and used to construct alpha triangulations via the *alphaTriangulation* function.

To build the retina’s triangular mesh, we implemented a custom region-growing algorithm. Starting from a manually selected triangle over a bristle cell complex (*initial triangle*), the algorithm iteratively added non-adjacent triangles until completion of a *valid triangle* set. The remaining triangles were classified as *inverse triangles*. Surface area-based color coding was applied to each triangle.

For *talin-IR* retinas (*GMR-Gal4>talin-RNAi*), the segmentation procedure differed from that employed for the wild-type *D. melanogaster* (“WT”) and *D*. *mauritania* (“Mau”), since grommets were largely absent, precluding neural network segmentation. These samples were fully segmented manually by delineating boundaries in *Volume Segmenter*. Boundary coordinates were extracted via custom scripts and used in downstream analyses.

### Building the different simulations

#### sWT

To make any triangulation, in the case of the one defined by the set of valid triangles of WT, sWT, compatible with the FEM computational model employed in this study^21,58,59^(Supplementary Methods), several preprocessing steps are required:

1. *Quadratic triangulation*: we converted the initial triangulation into a quadratic triangulation using a custom MATLAB routine. Each triangle was redefined with six nodes, three at the vertices and three at edge points, yielding two matrices, a *coordinate matrix* (*coordinates*) of size and a *connectivity matrix* (*elements*) with nodes ordered clockwise^60^.
2. *Dirichlet boundary conditions*: we imposed these conditions by identifying the boundary nodes using MATLAB’s *boundary* functions and fixing all their *degrees of freedom* (dofs) to zero. These include three translational displacements along the *x*, *y*, *z* directions (dof = 1, 2, 3) and two rotational displacements about the *x* and *y* axes (dof = 4, 5). The conditions were stored in the *fixnodes* matrix to ensure model stability near boundaries.
3. *Nodal points*: the external forces were defined for internal (non-boundary) nodes, recorded in the *pointload* matrix. Initially, forces were applied in the *z*-direction, later updated during iterations to acto normal to each triangle’s surface (Supplementary Methods).

#### sWTi

To assess whether the gradient in the triangular pattern encodes the three-dimensional curvature of WT, we generated an inverted control pattern, referred to as sWTi, defined by the set of inverse triangles (Supplementary Fig. 3f, g). Once it was established, the same analytical and computational procedures were applied as used for the sWT model.

#### sIT

For each of the sWTs, a corresponding uniform triangulation, sIT (Fig. 2d), was generated to create a mesh without spatial gradients in triangle size while preserving the original geometric pattern of valid triangles.

The process began by extracting the sWT boundary points and constructing a *uniform lattice* in the $x-y$ plane. This lattice consisted of two alternating lines of points spaced by the average base ${d}_{b}$ and height ${d}_{h}$ of sWT triangles, producing congruent triangles of eqaul área across the domain. Points within the sWT boundary were selected and triangulated similarly tos WT methods, forming the sIT mesh.

Since the number of triangles in sIT could differ from sWT, an iterative adjustment of ${d}_{b}$ and ${d}_{h}$ was performed until the number of triangles between sWT and sIT is reduced to within 5% (Supplementary Data 3).

#### sRub

For each triangulation based on the valid triangle set sWT, a corresponding rubber-like triangulation, sRub (Supplementary Fig. 2h), was generated to create a spatially continuous, non-structured and homogeneous mesh. Unlike the structured uniform triangulations (sIT), sRub was created using a simpler metho leveraging MATLAB’s Partial differential equation toolbox to automatically generate scrambled triangles within the sWT boundary. As a result, sRub meshes contained significantly more triangles than sWT, increasing computational demands. To balance simulation accuracy and efficiency, edge lengths were constrained between ${H}_{\max }=10\mu m$ and ${H}_{\min }=5\mu m$ (Supplementary Data 3).

#### sTalin

For the sTalin models, the same meshing procedure described for the sRub models was applied, including the specification of the maximum and minimum target edge lengths, ${H}_{\max }$ and ${H}_{\min }$, respectively (Supplementary Fig. 6f). However, in this case, rather than utilizing the boundary of the corresponding sWT model, the triangulation was generated using the manually defined boundary of the *talin-RI* pupae.

#### sMmp2

The same procedure described for the sTalin models was applied to *Mmp2* samples.

#### sMau

The same procedure described for the sWT models was applied to Mau samples (Fig. 2i).

#### sMau*

In the case of the sMau* models, it was necessary to refine the set of valid triangles of Mau samples due to the disruption of the characteristic triangulation pattern in specific regions of the anterior part of the retina (Supplementary Fig. 5f). This disruption resulted in the inclusion of computational triangles that did not accurately represent the basal surface of *Drosophila mauritiana* pupae. These non-representative triangles, which represent on average the 3% of the total triangles (Supplementary Data 3), were systematically identified based on morphological discrepancies with the expected basal surface geometry and subsequently excluded from the valid sMau triangulation set using a custom MATLAB script.

#### sGigasT

The same procedure described for the sWT models was applied to the gigas samples (Fig. 3f).

#### sGigas

In the case of the sGigas models, it was necessary to manually curate the set of valid triangles of gigas samples due to the disruption of the characteristic triangular pattern in the dorsal part of the retina (Fig. 3). This disruption meant that some of the triangles included in the sGigasT pattern were not present and therefore the direct triangulation of the grommets did not accurately represent the pattern of basal triangles of *gigas-IR* pupal retinas. These extra triangles, which represented on average 25% of the dorsal triangles (Supplementary Data 3), were systematically identified based on morphological discrepancies with the expected basal surface geometry and subsequently excluded from the sGigasT triangulation set using a custom MATLAB script to generate the sGigas triangular patterns.

### 3D adult eye image segmentation and postprocessing

To segment adult eyes independently from the heads across genotypes, we used the *Volume Segmenter* application with image data imported via a custom MATLAB script. Manual segmentation was performed, followed by linear interpolation. The resulting label volumes were binarized and cropped for individual eye processing. A custom MATLAB algorithm extracted the apical surface by converting the segmented volume into a calibrated 3D point cloud. The point cloud was rotated so that the dorso-ventral axis lay in the $x-y$ plane, aligning the apex (the point with the highest $z$-coordinate) normal with the $z$-axis. Spatial density was enhanced through interpolation with *griddata* function. The eye boundary was determined by projecting the point cloud onto the $x-y$ plane and identifying its outer contour. Starting from the apex, the surface was propagated through connected points until the boundary was reached. Final boundary points were added and re-interpolated to yield a continuous apical surface.

Although the apical surface of the eye can be extracted from the segmented volume, the resulting representation is not suitable for the computation of dimensionless Gaussian curvature^61–63^.

To address these limitations, we developed a dedicated MATLAB algorithm that consists of:

1. *Point cloud downsampling*: as proved in ref. ^64^ the dimensionless Gaussian curvature depends on the distance between a point and its neighbors. A downsampling strategy based on *farthest point sampling* (FPS)^65^ was implemented to iteratively select the subset of points that maximized spatial coverage while preserving the surface's overall structure.
2. *Surface smoothing*: to refine the mesh and reduce noise in the point cloud, we apply a smoothing operation based on Laplacian smoothing, which operates by iteratively adjusting the vertex positions to achieve a smoother surface while preserving the overall geometry. The smoothing process is performed as follows:
  2.1. *Adjacency matrix construction*: at first, the adjacency matrix $A\in {{\mathbb{R}}}^{N\times N}$ is built. Given the set of vertices $V$ and faces $K$ connectivity of the mesh (Supplementary Methods), for each triangular face $\{i,j,k\}\in K$, the adjacency matrix satisfies that $A(i,j)=A(j,i)=1$.
  2.2. *Laplacian matrix computation*: the Laplacian matrix $L$ is computed using the relation $L=D-A$, where $D$ is the degree matrix, defined as $D={diag}\left(A\cdot {{{\boldsymbol{1}}}}\right)$, where ${{{\boldsymbol{1}}}}$ is a column vector of ones.
  2.3. *Iterative smoothing*: the vertex positions are iteratively updated using the explicit smoothing scheme:
    1 $${V}^{\left(t+1\right)}={V}^{\left(t\right)}-\lambda L{V}^{\left(t\right)}$$
    where $\lambda$ is the smoothing factor, and $t$ denotes the iteration index. A smoothing factor of 0.1 was chosen, and a total of 25 iterations are performed. This pair of values were chosen to balance convergence and preservation of geometric features, since small $\lambda$ implies that each iteration produces modest changes in vertex positions, thereby avoiding excessive smoothing or distortion.
  2.4. *Isotropic explicit remeshing*: irregular meshes can introduce significant local distortions in curvature computation^66^. To address this issue, an isotropic explicit remeshing step is implemented using *PyMeshLab*^67^. This remeshing algorithm is based on an energy minimization procedure, and only the number of iterations is needed. We set the number of iterations to 25, which is sufficient to reach convergence of the remeshing energy minimum.

### Measuring dimensionless Gaussian curvature

The set of angles formed at vertex ${v}_{i}$, by each of the incident triangles, ${d}_{i}$, on it is denoted as $\left\{{\alpha }_{1}^{i},{\alpha }_{2}^{i},...,{\alpha }_{{d}_{i}}^{i}\right\}$, where each ${\alpha }_{j}^{i}$ represents the internal angle at ${v}_{i}$ within the $j$-the incident triangle. According to refs. ^62,64,66^, the dimensionless Gaussian curvature ${G}_{i}$ over a neighborhood of vertex ${v}_{i}$ can be approximated by a discrete formulation:

2 $${G}_{i}=2\pi -{\sum }_{j=1}^{j={d}_{i}}{\alpha }_{j}^{i}.$$

This formulation may yield misleading results due to inherent geometric and topological characteristics of the mesh, such as open boundaries or topological defects. For vertices associated with such irregularities, defined as *degenerate vertices*, the computed values of ${G}_{i}$ does not reflect the curvature of the surface. These degenerate vertices are identified and removed using a custom-developed MATLAB algorithm:

1. *Detection of boundary edges and vertices*: edges that appear only once in the edge incidence map are classified as *boundary edges* and its vertices as *boundary vertices*. To improve curvature accuracy, all vertices in triangles containing any boundary vertex are also excluded from curvature calculations.
2. Degeneracy filtering based on geometric and angular criteria: to ensure the integrity of curvature values, additional geometric criteria are enforced to identify degenerate vertices:
  2.1. According to refs. ^68,69^ vertices with any incident angle below 30° or above 90° may introduce a bias in ${G}_{i}$. Although the isotropic explicit remeshing algorithm was applied, occasional violations still occur in regions near the boundary. To account for numerical accuracy, vertices with any incident angle below 33° or above 87° are identified as degenerate vertices.
  2.2. In pupal samples, certain vertices located near the boundary may escape detection in the preceding filtering steps. Although such instances are infrequent, these vertices exhibit geometric characteristics analogous to true boundary points. For that reason, vertices for which $\left|{G}_{i}\right| > 0.01$ are also identified as degenerate vertices.
3. Identification of interior vertices: vertices not marked as boundary-related in the previous step are designated as interior vertices. These are the only candidates considered for reliable Gaussian curvature estimation.

### Shape similarity metric, the Gaussian metric

According to refs. ^70,71^ it is possible to define a similarity measure between two geometric models by means of a distance function $d(A,B)$, that must satisfy the following properties:

- Non-negativity, $$d(A.B)\ge 0$$.
- Symmetry, $$d(A,B)=d(B,A)$$.
- Smaller values of $d(A,B)$, more similar the shapes of $A$ and $B$.

As demonstrated in refs. ^63,71^, the integral of ${G}_{i}$ remains invariant under geometric transformations, so its distribution inherently defines a robust distance function for shape similarity.

As described in refs. ^63^, the computation of that distribution is obtained by projecting the mesh vertices onto the $x-y$ plane and transforming the point cloud, along ${G}_{i}$ values, into polar coordinates $\left({r}_{i},{\theta }_{i}\right)$, where the origin is defined as the geometric center of the point cloud $\left({x}_{c},{y}_{c}\right)$ and the radial distance is computed as ${r}_{i}=\sqrt{{\left({x}_{i}-{x}_{c}\right)}^{2}+{\left({y}_{i}-{y}_{c}\right)}^{2}}$.

The vertices are systematically ordered according to their radial distances ${r}_{i}$. To partition the vertex set into $n$ concentric regions, we define the ${j}^{{th}}$ region as comprising those vertices whose radial coordinates satisfy ${r}_{i}\in [{r}_{j},{r}_{j+1}),$ where ${r}_{j}$ denotes the $\left(\frac{100\left(j-1\right)}{n}+1\right)$ percentile of the distribution $\left\{{r}_{I}\right\}$. This procedure yields $n$ distinct groups of vertices, denoted as ${\left\{{v}_{i}\right\}}_{i\in {I}_{1}},{\left\{{v}_{i}\right\}}_{i\in {I}_{2}},...,{\left\{{v}_{i}\right\}}_{i\in {I}_{n}}$, where the index set ${I}_{j}$ identifies all vertices belonging to the ${j}^{{th}}$ radial group. By construction, each group contains an equal number of vertices.

For each region ${I}_{j}$, the integrated dimensionless Gaussian curvature ${G}_{j}^{T}$, called *Gaussian curvature profile*, is computed as follows:

3 $${G}_{j}^{T}={\sum }_{i\in {I}_{j}}{G}_{i}$$

A vector ${{{\boldsymbol{G}}}}=\left[{G}_{1}^{T},{G}_{2}^{T},...,{G}_{n}^{T}\right]$ is constructed that represents the surface. Consequently, the problem of comparing original surface meshes is reduced to the comparison of their ${{{\boldsymbol{G}}}}$ vectors (Box 1). To this end, a distance function ${d}_{G}$ is defined as follows:

4 $${d}_{G}\left({G}_{1},{G}_{2}\right)=1-\left|r\left({G}_{1},{G}_{2}\right)\right|$$

where ${d}_{G}\in [{{\mathrm{0,1}}})$ and $\left|r\left(G1,G2\right)\right|$ is the absolute value of the Pearson correlation coefficient defined as:

5 $$r\left({G}_{1},{G}_{2}\right)=\frac{{{\mathrm{cov}}}\left({G}_{1},{G}_{2}\right)}{{\sigma }_{1}{\sigma }_{2}}$$

The distance function ${d}_{G}$ will henceforth be referred to as the *Gaussian metric*. Values near zero indicate high shape similarity between meshes, while values near one indicate significant shape dissimilarity (Box 1). According to ref. ^63^, $n\in \left[{{\mathrm{5,10}}}\right]$, so we have adopted $n=10$ to maximize the sensitivity of the Gaussian metric.

### Matching adult size and selecting the appropriate iteration

Following completion of all simulations generated by the computational model, 1000 for sWT, sWTi, sIT, sMau and sMau*, and 2000 for sRub and sTalin, the resulting meshes were postprocessed using the same procedure described for adult specimens (Methods, 3D adult eyes image acquisition, segmentation and postprocessing). In this context, 75 iterations of the mesh smoothing algorithm were applied. This number was chosen to accommodate the fixed boundary conditions, which impose geometric constraints on curvature near the boundary and within regions that must remain free from negative curvature or irregular meshing artifacts. Given that the employed smoothing technique relies on the adjacency matrix, it enables selective regularization: boundary-adjacent regions are smoothed appropriately, while curvature in unaffected areas is preserved, thereby preventing excessive flattening due to oversmoothing.

Among the resulting iterations, it is necessary to select the one that best approximates the morphology of the adult specimens corresponding to the given genotype. This selection process is non-trivial, as the minimum of the Gaussian metric is not guaranteed to be unique, nor does it necessarily yield a configuration that accurately reflects adult morphology (Supplementary Fig. 4c). This limitation arises because the Gaussian metric, based on the distribution of dimensionless Gaussian curvature, does not account for absolute size.

To incorporate both shape and scale, ensuring that the selected iteration reflects not only the curvature distribution but also the physical size of the adult specimens, the following criteria were established to guide the selection of the optimal iteration:

1. *Size criterion*: to account for adult size, we first assessed whether significant differences existed between the lengths of the major axis in adults and pupae of the corresponding genotype (Supplementary Data 2 and 3). As no statistically significant differences were observed in either case (Supplementary Data 6), the depth of the raw adults (preprocessing ones) was defined as the difference between the mean depth of the three-dimensional boundary and the depth of the deepest point. For each genotype, the acceptable depth range was defined as the mean adult depth ± one standard deviation. Any simulated specimen whose depth fell within this interval was considered to exhibit a depth-to-axis ratio statistically indistinguishable from that of the adults, thereby satisfying the size criterion (Supplementary Fig. 4b).
2. *Gaussian metric criterion*: for each raw iteration of the simulations (Supplementary Fig. 4a), depth was computed as the maximum value of the z-coordinate, given that the boundary was constrained to *z* = 0. Additionally, G was calculated, along with $< {d}_{G} >$, defined as the mean Gaussian metric from the distribution of adult individuals of the same genotype. This procedure yielded a pair (depth,$< {d}_{G} >$) for each iteration. Among the iterations whose depths fell within the genotype-specific confidence interval defined by the size criterion, the one exhibiting the minimum Gaussian metric was selected (Supplementary Fig. 4c), thereby ensuring both geometric fidelity and curvature-based similarity to the adult morphology.

### Sampling in vivo retinas

The same image-processing pipeline used for the 42-h APF retinas was applied to the *Ecad::GFP* in vivo images (see “Antibody staining and imaging section”). Briefly, grommets were segmented, after which they were used as triangle vertices. The resulting meshes were triangulated, and the area of each triangle was computed (Supplementary Fig. 2). The procedure used to identify and select the apical and basal regions was likewise applied to the 42-h APF retinas. Following cropping of these regions, triangle areas were extracted for subsequent analysis (Supplementary Fig. 2).

### Hexagonality measurements of ommatidia

The same methodology described in ref. ^72^ was applied to the images of the apical side of WT and *talin-IR* pupal retinas stained with Ecadh (Supplementary Fig. 6). In each image, the dorsal–ventral (vertical) and anterior–posterior (horizontal) axes were identified. For each ommatidium, the ratio between the vertical and horizontal distances from the centroid of the cone cell cluster to neighboring ommatidia was computed and used for subsequent statistical analysis.

### Reporting summary

Further information on research design is available in the Nature Portfolio Reporting Summary linked to this article.

## Supplementary information

Supplementary Information

Description of Additional Supplementary Files

Supplementary Data 1

Supplementary Data 2

Supplementary Data 3

Supplementary Data 4

Supplementary Data 5

Supplementary Data 6

Supplementary Movie 1

Supplementary Movie 2

Reporting Summary

Transparent Peer Review File

## Data Availability

All data used in this study is freely available as a supplement to this manuscript (Supplementary Data). The individual raw images are available upon reasonable request.

## Code availability

Code is available at: https://github.com/ComplexOrganizationOfLivingMatter/DrosophilaEyeCurvature.
