# Supplementary material for: A natural programmable metamaterial controls 3D curvature of compound eyes
Source: Nat Commun. 2026 Jun 30;17:5553. doi: 10.1038/s41467-026-74276-6 (PMC13319490; doi:10.1038/s41467-026-74276-6)
Supplement: Supplementary file 2 — Description of Additional Supplementary Files [file 41467_2026_74276_MOESM2_ESM.pdf]

Description of Additional Supplementary Files

File name: Supplementary Data 1
Description: Gaussian metric pairwise values of Box.

File name: Supplementary Data 2
Description: Geometric features of adult eyes: Values for the geometric features extracted from the segmented adult eyes.

File name: Supplementary Data 3
Description: Geometric features of simulations: Values for a list of features of the inflated simulations. The features are: Number of triangles, Iteration to match the adult, Depth, Major Axis, Minor Axis, Triangle Mean Area, Standard deviation of Triangle Area, Triangle Mean Height, Triangle Mean Edge Length, Standard deviation of Triangle Edge Length, Triangle Ratio Thickness-Height.

File name: Supplementary Data 4
Description: Gaussian metric pairwise values of adult eyes and simulations.

File name: Supplementary Data 5
Description: Statistics of Gaussian Metric distributions: Information about the statistical tests used to quantify the differences in the performed comparisons of the Gaussian metric distributions. The type of test and the corresponding p-value are included.

File name: Supplementary Data 6
Description: Statistics of Geometric Features distributions: Statistical tests used to compare geometric feature distributions along with test types and p-values, are provided. The features are: Major Axis and Depth.

File name: Supplementary Movie 1
Description: 360º View of the segmented surface of an adult *Drosophila melanogaster* eye. Video of the segmented eye surface shown in Fig. 2b rotating 360º to display its 3D structure from multiple angles.

File name: Supplementary Movie 2
Description: Inflation of a 2D triangle mesh to reach sWT. Video of the inflation process of the 2D triangle mesh shown in Fig. 2c to generate the sWT 3D surface.
