# Supplementary material for: A natural programmable metamaterial controls 3D curvature of compound eyes
Source: Nat Commun. 2026 Jun 30;17:5553. doi: 10.1038/s41467-026-74276-6 (PMC13319490; doi:10.1038/s41467-026-74276-6)
Supplement: Supplementary file 12 — Transparent Peer Review File [file 41467_2026_74276_MOESM12_ESM.pdf]

nature portfolio

Peer Review File

A Natural Programmable Metamaterial Controls 3D Curvature of Compound Eyes

Corresponding Author: Dr Luis Escudero

This file contains all reviewer reports in order by version, followed by all author rebuttals in order by version.

Version 0:

Reviewer comments:

Reviewer #1

(Remarks to the Author)
This manuscript presents an original and elegant study exploring how the three-dimensional curvature of the Drosophila compound eye is generated during development. The authors identify a supracellular triangular mesh at the basal surface of the retina and propose that this mesoscale organization acts as a natural programmable metamaterial that encodes local curvature. By integrating quantitative imaging, comparative analyses between Drosophila melanogaster and Drosophila mauritiana, genetic perturbation using Talin RNAi, and computational modeling, the work brings together multiple approaches to address how cellular architecture translates into precise organ shape. The concept that mesoscale geometry can determine macroscopic form is both compelling and interdisciplinary, bridging developmental biology, physics, and materials science. Applying a metamaterial framework to living tissue is particularly innovative and may open a new avenue for understanding morphogenetic control. Overall, this is a creative and potentially influential study, though several aspects would benefit from clarification and further support.

While the computational model reproduces the overall curvature convincingly, the physical and mechanical assumptions on which it rests are not sufficiently described. It is not clear what mechanical properties were assigned to the mesh elements, how the pressure or boundary conditions were applied, or whether the model assumes static equilibrium or allows for out-of-plane deformation. Because the authors conclude that only the triangular micropattern can reproduce the adult 3D curvature, readers need to understand what makes this particular pattern unique from a mechanical standpoint. A concise explanation of the key assumptions and physical reasoning—ideally in the main text rather than only in the Supplementary Methods—would make the modeling framework more transparent and easier to interpret.

The comparison between D. melanogaster and D. mauritiana provides interesting support for the link between the triangular mesh and curvature, but the statement that the mechanism is conserved “across Drosophila species” may be too strong. These two species are closely related and diverged relatively recently, so the data more appropriately demonstrate conservation between these two, rather than across the entire genus. Phrasing such as “may be conserved among Drosophila species” or “at least between D. melanogaster and D. mauritiana” would more accurately reflect the evidence. A short discussion of evolutionary context, or future inclusion of a more distantly related species, could strengthen this point.

The Talin RNAi experiments are important in linking basal adhesion to curvature robustness. However, because Talin has multiple functions beyond integrin-mediated attachment—including roles in cytoskeletal organization and cell polarity—it is difficult to exclude the possibility that the observed effects stem from broader cellular changes. The argument that the triangular mesh connectivity itself determines curvature would be more convincing if similar phenotypes were observed after perturbing other adhesion-related molecules (e.g., integrins, Kindlin, or ECM linkers such as Laminin). Demonstrating consistent outcomes via multiple genetic routes would provide stronger causal support for the proposed mechanism.

The fourth point is, in my view, the most critical issue in this study. The causal link between the triangular mesh and curvature remains somewhat indirect. The model provides a plausible mechanism, but the experimental data mainly show correlation rather than causation. Given that the central claim of the paper is that mesoscale geometry drives morphogenesis, a more direct test of this causal relationship would be essential to solidify the conclusion. While direct physical manipulation of the mesh may be technically difficult, indirect approaches could still be informative—for example, modulating hydrostatic pressure during pupal development, altering local tension optogenetically, or quantitatively comparing predicted and measured curvature gradients. If such experiments are not feasible at present, clearly stating that

causality is inferred from modeling, and discussing this limitation explicitly, would make the argument more balanced and transparent. In my view, strengthening this causal analysis is the key to consolidating the scientific impact of this study.

A related point concerns the claim that “the triangular mesh is critical for the robustness of the 3D curvature.” This assertion would be reinforced by showing that other mesoscale structures—such as apical cell patterning or the corneal lens layer—do not play comparable roles. Demonstrating that curvature remains intact when apical organization is perturbed but the basal mesh remains connected would provide compelling evidence that the basal structure specifically underlies robustness. If such experiments are impractical, the authors could still discuss why the basal mesh is most likely the principal determinant. This issue is conceptually linked to the previous point, as both address how the system ensures causal and structural specificity in curvature control.

(Remarks on code availability)
I checked the GitHub page, but the number of files was so large that I couldn’t follow the entire contents.

Reviewer #2

(Remarks to the Author)
In this study, Garrido-Garcia and colleagues describe an original morphogenesis model whereby prepattern of basal cell arrangement forming a network of connected structures encodes the 3D shape and curvature of an hemisphere upon inflation. This metamaterial provides a very relevant morphogenetic framework which applies very well to the morphogenesis of the compound eyes of Drosophila melanogaster and Drosophila mauritania, and can also explain the 3D shape upon perturbation of talin and the binding to ECM. The paper, relatively short, is mostly based on precise characterisation of the triangular pattern of the retina in the two species and upon perturbations of talin, and prediction of the emerging 3D curvature using argent based model as well as carefull quantitative comparison of the experimental and simulated 3D shape. The paper is original, well structured, easy to read, based on very clean (and non trivial) 3D characterisation and the results quite compelling. This is of course relatively correlative in many aspects, but it convincingly shows the relevance of the modelling framework. The results should be of wide interest in the field of morphogenesis and goes along the line of other recent results trying to dissect 3D morphogenesis in the framework of 2D deployable systems. I am overall very supportive for publication, but I do have few suggestions of few additions of experimental conditions that would help to strengthen the validity of the model as well as some clarification of the biological system and potential limitation/hypothesis in the model.

Suggestions :

1. The model convincingly manages to recapitulate D melanogaster, D Mauritania and talin depletion eye shape. However all these perturbations/genetic background relie on global effect that might be quite pleiotropic. Assuming that the local triangular pattern mostly have a local impact on curvature, it would be very relevant to include some clonal/local perturbations to see whether this leads to predictive local curvature changes. This might be difficult with clonal tools if it is not possible to image the triangular pattern/clones and the 3D eye shape in the same tissue (although this might be possible at late pupal stage ?), but it could be done at least using driver with predictive pattern affecting only a portion of the eye (e.g. : mirror-gal4).

2. So far the only perturbative condition provided is based on Talin depletion. The results are really convincing and the increase of 3D curvature tissue to tissue variability really compelling. Yet, talin may affect other parameters that could affect 3D tissue shape (e.g. : basal membrane tension, lateral tension, Rho activity, polarity…). To fully demonstrate that the triangular pattern plays an instructive role, it would be good to provide at least one alternative perturbative condition affecting the pattern and 3D curvature. For instance, would it be possible to affect the apex of triangles (the grommet) for instance by affecting locally IOC fate ?

3. The fact that triangle pattern is enough to recapitulate the 3D shape is not exclusive of an inverse relationship (3D shape imposing the triangle pattern). The image describing triangular pattern seems to emerge pretty late (around 40h APF) while the inflation/curving of the eye starts around 20h APF. To which extend the curving itself is influencing the triangular pattern ? The author should clarify this and clearly document whether the basal pattern is fixed prior to curving/inflation.

4. If I am correct the metamaterial model assumes constancy of the material property of its unit (here the triangle). However I suspect that depending on tissue specific localisation, the quantity of ECM/actin may vary and the tensional state of triangles may not be totally homogeneous. Of course, this should be reflected in the shape/size of triangle that the authors can capture in their model and experimental data. Yet, I think it would be important to openly discuss this important reductionist hypothesis and its relevance to describe the system (at least in the discussion).

Other minor points :

1. The concept presented in this article are not totally different from a recent study from Natalie Dye group (https://www.science.org/doi/10.1126/sciadv.adp0860) using the concept of programmable/deployable 2D system to from complex 3D structure (applied to the wing disc). I do acknowledge that the modelling framework and concepts are different (in their case using in plane gradient of strain), but both studies use the concept of programming 3D shape in a 2D pattern. It would be relevant to include this reference in the discussion.

2. Figure Box d: Could the authors be more specific about the parameter calculated in the values on the arrows between

conditions/simulation steps ? The info can be found in methods, but it would help to quickly describe this in the legend.

(Remarks on code availability)
I didn't review the code and cannot comment this section.

Reviewer #3

(Remarks to the Author)
Reviewer Summary:
This paper by Garrido-Garcia et al. investigates how the basal cellular pattern of the Drosophila pupal retinal epithelium influences regional anisotropies in adult retinal curvature. Specifically the paper considers how isotropic pressure, exerted on a non-uniform triangular "mesh", might induce a 3-D morphology with an anisotropic distribution of local curvatures. They implement a mathematical framework that allows them to apply pressure to a 2-D mesh with specific geometry and then examine the resulting 3-D shapes using a Gaussian curvature metric. Using a 2-D mesh that matches the network of secondary pigment cell (PC) basal contacts in a wildtype pupal retina, they show that pressure-induced inflation of this flat surface results in a half-dome structure that recapitulates the regional curvature anisotropies observed in adult fly eyes. Disruption of basal pattern via RNAi knockdown of the integrin associated protein Talin results in regional curvature defects in adult eyes and in matching simulations. They conclude that tissue-scale basal pattern in the pupal retina critically informs regional curvature anisotropies in the adult eye. Overall, the question of how specific cellular morphologies channel forces from the growth environment to produce functional organ morphologies is of broad general interest. Further, the modeling framework is a clever way to address tissue properties and relationships that are hard to measure and manipulate in vivo. Thus, the work has the potential to be of interest outside of the fly eye field.
However, I have concerns about the approach and some of the conclusions, and so do not recommend publication of the manuscript in its current form. Of greatest concern, the main premise of the modeling experiments does not match the known in vivo developmental progression of the retina. Second, the limited in vivo validation of concepts learned from the computational modeling contributes to a general tendency to over-interpret experimental results and to overstate what should be more speculative conclusions. A third important point is that throughout the text, the general term "curvature" is used to mean both the organ-scale curvature of the eye and the finer-scale anisotropies in local curvature – using language that clarifies more precisely these different concepts and scales and that distinguishes between curvature acquisition, refinement and maintenance, is essential to improving the accuracy with which the data are represented, interpreted and discussed.

Major comments:
1. The mathematical modeling simulates the acquisition of 3-D curvature by applying pressure to an initially flat 2-D surface to induce out of plane bending. The input is a patterned 2-D surface with a triangular mesh derived from the basal pattern of secondary PC contacts typical of a 42h APF retina. However, recent work from the lab of one of the corresponding authors of this study shows that by 42h APF the tissue has already acquired its organ-scale 3-D curvature (Lancaster et al. doi: 10.1016/j.cub.2025.05.048). Indeed the Garrido-Garcia et al. manuscript references this prior paper and states "the thin pupal retinal epithelium becomes curved around 20 hours after pupa formation (hAPF), under the action of hydrostatic pressure". Compounding the model vs. biology discrepancy, another previous paper from the same group (Walther et al. doi: 10.1371/journal.pbio.3002783) shows that at ~20-24h APF, stereotyped basal pattern has not yet been established and that PC basal contacts are highly disordered (these observations corroborate those reported in many earlier papers, starting with Longley & Ready 1995 (doi: 10.1006/dbio.1995.1292)). Therefore, the central premise of the modeling experiments and of the paper overall, that pressure acts on a flat 2-D surface with a specific, stereotyped pattern does not match the developmental progression of retinal basal patterning and curvature acquisition seen in vivo. This discrepancy calls into question the validity of the study as currently framed.
It seems formally possible that basal pattern (which is acquired after tissue-scale curvature establishment, according to the timeline presented in Fig 1E and previous work from multiple labs that have characterized basal pattern formation) refines pre-established retinal curvature and is responsible for the local curvature anisotropies in adult eyes as proposed. By performing similar tissue-scale Gaussian curvature measurements on pupal retinas both before and after basal pattern refines (24h vs 42h APF) the authors could test this. If basal patterning refines tissue curvature, the prediction is that curved retinas at 24h APF would display gaussian curvature metrics that differ from those of adult eyes, while 42h APF retinas would not. The computational model would also need significant revision to incorporate the initially disordered basal pattern (known to be present in the flat tissue) and the subsequent acquisition of the basal triangular mesh in an already curved surface. Such experiments would allow the authors to define more clearly the principal question and conclusion of their study, as the resultant data would provide evidence that basal pattern is required for regional curvature refinement but does not influence organ-scale curvature acquisition. This interpretation would also be consistent with the result that Talin RNAi retinas (which have perturbed basal pattern) produce local curvature anisotropy defects, but have an intact tissue-scale "half-dome" structure.
Another formal possibility that might reconcile the current disconnect between model and biology is that the influence of internal hydrostatic pressure might only become relevant after the basal network pattern has refined. However to my knowledge this is not known and so can't be assumed, and testing it experimentally seems challenging.

2. The authors state two ideas central to their proposed model as accepted facts. In my opinion, there is insufficient experimental validation to make these claims so strongly.
First, the authors state that pressure induces curvature, and cite Lancaster et al., 2025. To ask if positive pressure within the developing pupa influences retinal curvature, Lancaster et al. punctured the pupal case distal to the head capsule, and noted tissue-scale defects in retinal curvature. There is not sufficient methodological detail provided to fully understand the

experimental protocol or the developmental stage at which the puncture was performed, but visually it appears it was made at a stage of development when curvature has already been acquired; if so, then puncturing the pupal case perturbs established curvature. Therefore from this experiment alone, the only non-speculative conclusion is that pressure is required for curvature maintenance. It is interesting and appropriate to speculate the pressure might induce curvature, and might be required to refine local curvature while maintaining tissue scale form. Since measuring dynamic changes in developing pupae is technically challenging, the approach of exploring the hypothesis that pressure induces curvature with a computational model is appropriate, but the authors need to adjust the language throughout the manuscript to acknowledge the speculative nature of proposed models and to acknowledge that future in vivo experiments will be needed to validate the predicted biology.
Second, in the introduction the authors describe the crystalline lens as a “hard polymer” and state that its secretion locks the organ scale curvature in place and prevents any further morphological changes. While this seems plausible, neither experimental validation nor references are provided. To my knowledge there is only speculation in the literature on these points. Normally I would recommend simply removing this statement, but the idea is important to the proposed model. Specifically the authors propose that the initial triangular pattern of secondary PC basal feet establishes early in development the curvature anisotropies observed later in the adult, and that the crystalline lens locks in these early regional differences in curvature throughout subsequent phases of development when the IOC basal pattern changes substantially. At a minimum, the authors need to confirm in vivo that the curvature anisotropies seen in the adult eye are present at a stage in pupal retinal development where basal pattern of the retina is established, but the vitreous lens has not been secreted (~40h APF). In other words, they need to define exactly when the fine-scale curvature differences seen in the adult are actually established in the developing retina.

3. Related to both concerns discussed above in points 1 and 2, there is a tendency throughout the manuscript to make overly general statements and to over-interpret experimental results without the necessary in vivo validation. Therefore, claims made in this paper need to be tempered to more accurately reflect conclusions appropriate to the data presented. The discrepancy in the timing of tissue-scale curvature acquisition and basal pattern formation underlies the most serious examples. For example, the discussion states that this paper uncovers a “novel strategy to program a 3D shape in vivo: uniform hydrostatic pressure acts on a patterned non-homogeneous tissue.” I also found it inappropriate to claim discovery of a “new level of organization” at the basal surface. The pattern of 2oPC feet has long been noted; what is new and exciting in this study is the idea of considering it as a 2-D patterned mesh, or metamaterial, that templates 3-D tissue properties.

Minor comments:
1. The authors argue that in order for the basal pattern to be considered a meta-material that regulates retinal curvature, it must be stereotyped. Presumably the authors mean that this pattern should be present with little variability across a population of retinas? In the supplemental materials, they show retinas from one male and one female fly, and the two triangular meshes do appear similar. However, it seems important to account for population scale variability, and so triangular mesh structures should be segmented from a larger population of animals and a statistical analysis of the spatial variability should be calculated. This average mesh structure should then be used in the simulations.
2. The authors segment a planar (2-D) triangular mesh pattern from 42hr APF retinas. As stated in this paper and by Lancaster et al. 2025, these retinas have already acquired a stable tissue-scale curvature. Additional methodological detail is needed to understand how they segmented the planar mesh from this curved surface. If 42hr APF retinas were flattened to image basal gromets and segment the triangular mesh structure, control experiments demonstrating that flattening does not perturb or distort the PC shapes need to be included. As spatial features of the pattern are critical for the curvature that the computational model generates (although not actually present in vivo when the curvature is acquired) clarification of this point is important.
3. The authors use RNAi-mediated knockdown of Talin to perturb basal pattern, and find regional curvature anisotropy defects in both the computational model and in adult retinas. Talin provides a mechanical link between cytoskeletal structures in the cell interior and integrin complexes that interact with extracellular matrix (ECM) structures. It has been demonstrated in the developing fly retina that cell-ECM interactions form a network with contractile properties at the basal surface of the retinal epithelium (Ready & Chang, 2023, doi: 10.1016/j.cub.2023.04.066). Thus Talin knockdown might also disrupt basal cell mechanics (in addition to basal pattern). Given this possibility, the conclusion that basal patterning defects induced by Talin knockdown are the sole driver of the resultant curvature anisotropy defects is too strong a statement.
4. The use of the Gaussian curvature metric is prominent throughout the paper, but the details of how it captures the regional differences in tissue curvature that this study focuses on are not sufficiently clear. To facilitate broader readability, this method should be explained in greater detail, focusing on how this mathematical concept is employed and how it captures regional curvature features of the retina. Some statistical comparison of model output to in vivo measurement should be included.
5. It looks to me like apical geometry in the Talin knockdown is altered, contrary to what is stated. Specifically the ommatidia appear more diamond shaped rather than hexagonal shaped as they are in the wildtype image in 1G. Perhaps the selected Talin RNAi image in 3B is not representative of the result? Statistical comparisons of apical geometry should be performed, and the selected image (and statement in the text) should reflect the results.
6. Justification (or mention of potential caveats) for using GMR-Gal4 to drives Talin RNAi in all retinal cells when the focus is on the secondary PCs should be added.

(Remarks on code availability)

Version 1:

Reviewer comments:

Reviewer #1

(Remarks to the Author)
I remain interested in the study and, overall, I am supportive of publication. The concept of a “natural programmable metamaterial” is both original and potentially important, and the authors have clearly made a substantial effort to respond to the reviewers’ comments. In particular, the addition of the gigas and Mmp2 experiments has strengthened the revision.

That said, I still have a few concerns that should be addressed before the manuscript is accepted. These concern, above all, the physical interpretation of the model and the strength of some of the claims drawn from it.

1. The revised manuscript does provide more technical information about the FEM model, including clarification of the t/h ratio and the use of thin-shell theory. However, my main concern from the previous round remains. The model is built on the assumption that the basal meshwork is the principal structure supporting tissue mechanics and bearing hydrostatic load, but this point has still not been demonstrated directly. At present, the manuscript does not provide direct experimental evidence that this multicellular basal mesh—rather than other components, such as the broader ECM or the cell bodies—is the main load-bearing structure in vivo. The simulations are useful and internally consistent, and I find them valuable as a theoretical framework. Still, without some physical measurement of the tissue mechanics—for example by AFM, micromanipulation, or laser ablation—it is difficult to know whether the assumed material properties of these triangles, and their proposed mechanical role, correspond to the actual biological system. At minimum, I think the manuscript should state this more explicitly and present the structural assumptions of the model as hypotheses that still require experimental validation.

2. The gigas RNAi experiments are also a welcome addition, since they address one of the key issues raised in the previous round. They provide an important functional test. Even so, I do not think they yet validate the full biological mechanism proposed by the authors. As presented, the simulations show that the geometric pattern, even under uniform material properties, is sufficient to reproduce the observed curvature. That is an important result. But it is not the same as showing that the pattern is the necessary cause, or even the dominant cause, in the living tissue. The authors themselves acknowledge that other factors—such as spatial differences in contractility or ECM composition—may also contribute in vivo. For that reason, I would encourage more restrained language here. The pattern may well act as a major guiding factor, or as a geometric template that works together with other mechanical properties, but the current data do not justify presenting it as the dominant cause.

3. I also still see a mismatch between the revised Discussion and the Abstract. In response to my previous Major Suggestion 5, the authors appropriately softened the interpretation in the Discussion and now describe the basal mesh as a “major mechanism” rather than the sole determinant. However, the Abstract continues to state that the findings “identify a previously unknown mechanism of morphogenesis control whereby supracellular 2D patterning endows a tissue with genetically programmable metamaterial properties to encode fine-scale 3D curvature with high precision.” This echoes Reviewer 3’s concern about overly deterministic language. In my view, this wording remains too strong. Saying that the patterning itself “endows” the tissue with these properties implies a degree of exclusivity and determinism that is not fully supported by the data, especially given the authors’ own acknowledgment that the mesh is unlikely to be the only relevant factor. I therefore ask that the Abstract be revised so that it matches the more measured tone now used in the Discussion and better reflects the multilayered nature of morphogenesis.

(Remarks on code availability)

Reviewer #2

(Remarks to the Author)
The authors have adressed all my concernes and significantly improved the manuscript, notably by providing novel perturbative conditions and using local perturbation. The thorough discussion about the timing of inflaction, basal pattern establishment and refinment of the curvature was also very information. I am now fully supportive for publication and would like to congratulate the authors for this very nice work.

(Remarks on code availability)

Reviewer #3

(Remarks to the Author)
The authors have done a very thorough job addressing reviewer comments. The new data, controls and text that have been added have greatly improved the rigor and thoughtfulness of manuscript. I have no further concerns and recommend publication.

(Remarks on code availability)

Open Access This Peer Review File is licensed under a Creative Commons Attribution 4.0 International License, which permits use, sharing, adaptation, distribution and reproduction in any medium or format, as long as you give appropriate credit to the original author(s) and the source, provide a link to the Creative Commons license, and indicate if changes were made.
In cases where reviewers are anonymous, credit should be given to 'Anonymous Referee' and the source.
The images or other third party material in this Peer Review File are included in the article's Creative Commons license, unless indicated otherwise in a credit line to the material. If material is not included in the article's Creative Commons license and your intended use is not permitted by statutory regulation or exceeds the permitted use, you will need to obtain permission directly from the copyright holder.
To view a copy of this license, visit https://creativecommons.org/licenses/by/4.0/

Point-by-Point response to the reviewers:

We thank the reviewers for their careful evaluation of our manuscript and for their constructive and insightful comments. Below, we provide a detailed, point-by-point response to each of their comments:

Overall opinion

This manuscript presents an original and elegant study exploring how the three-dimensional curvature of the Drosophila compound eye is generated during development. The authors identify a supracellular triangular mesh at the basal surface of the retina and propose that this mesoscale organization acts as a natural programmable metamaterial that encodes local curvature. By integrating quantitative imaging, comparative analyses between Drosophila melanogaster and Drosophila mauritiana, genetic perturbation using Talin RNAi, and computational modeling, the work brings together multiple approaches to address how cellular architecture translates into precise organ shape. The concept that mesoscale geometry can determine macroscopic form is both compelling and interdisciplinary, bridging developmental biology, physics, and materials science. Applying a metamaterial framework to living tissue is particularly innovative and may open a new avenue for understanding morphogenetic control. Overall, this is a creative and potentially influential study, though several aspects would benefit from clarification and further support.

R: We thank the reviewer for their positive evaluation of our work.

Major suggestions

1. While the computational model reproduces the overall curvature convincingly, the physical and mechanical assumptions on which it rests are not sufficiently described. It is not clear what mechanical properties were assigned to the mesh elements, how the pressure or boundary conditions were applied, or whether the model assumes static equilibrium or allows for out-of-plane deformation. Because the authors conclude that only the triangular micropattern can reproduce the adult 3D curvature, readers need to understand what makes this particular pattern unique from a mechanical standpoint. A concise explanation of the key assumptions and physical reasoning—ideally in the main text rather than only in the Supplementary Methods—would make the modeling framework more transparent and easier to interpret.

R1: To address this comment, we have revisited our description of the computational model, which now provides the information the reviewer was missing. This includes an explanation of mechanical deformation of triangles and how their interconnection is modeled. Specifically, the new paragraphs are:
Results, page 5: "The model was implemented as a finite element method (FEM)[21] to simulate the mechanical response of thin mesh structures using triangles as mesh units. This model is capable of capturing both membrane behaviors, responsible for in-plane area changes, and bending behaviors, responsible for out-of-plane curved deformations. Boundary conditions are imposed on the vertices at the perimeter by fixing all their degrees of freedom to zero, implying that these vertices cannot undergo any displacement or rotation (see Supplementary Methods). On the remaining vertices, a pressure load is applied along the normal direction, computed via an area-weighted averaging algorithm (see Supplementary Methods)."

Discussion, page 14: "The model captures these properties as follows. The material making the triangles is modeled as isotropic (i.e. with equal properties in all directions) and linearly elastic according to thin shell theory (Supplementary Data 3). The *t/h* ratio - with *t* being the thickness of the triangle sides, and *h* the triangle height- plays a fundamental role in the model, as it governs the relative stiffness of each of the triangles making up the mesh. Thus, variation in triangle area across the mesh allows this ratio to distribute mechanical forces differently according to the size pattern, such that curvature anisotropies emerge (as in "sWT"). In contrast, in the rubber-like material ("sRub") the *t/h* ratio is constant throughout the mesh precluding local curvature modulation."

2. The comparison between D. melanogaster and D. mauritiana provides interesting support for the link between the triangular mesh and curvature, but the statement that the mechanism is conserved "across Drosophila species" may be too strong. These two species are closely related and diverged relatively recently, so the data more appropriately demonstrate conservation between these two, rather than across the entire genus. Phrasing such as "may be conserved among Drosophila species" or "at least between D. melanogaster and D. mauritiana" would more accurately reflect the evidence. A short discussion of evolutionary context, or future inclusion of a more distantly related species, could strengthen this point.

R2: We have followed the reviewer's suggestion and have modified our text accordingly:
Results, page 8: "These results further indicate that during retinal development, the 2D patterned triangular mesh encodes the local curvature of the eye at least across the about 4 MYrs separating *D. melanogaster* and *D. mauritania*"
Discussion, page 15: "We have shown that *D. mauritania* shares with *D. melanogaster* the patterned basal triangular mesh. This fact suggests an evolutionary conservation of local curvature control. These two are related species separated by a relatively short evolutionary span. However, the organization of the compound eye is extremely conserved evolutionarily, not only within insects, but also in crustaceans which together are grouped under Tetraconata (denoting their shared ommatidial structure, which includes four cone cells)[43-45]. Therefore, we envisage that the mechanism for local curvature control we have described in related Drosophilas may be conserved well beyond flies, something that deserves further investigation."

3. The Talin RNAi experiments are important in linking basal adhesion to curvature robustness. However, because Talin has multiple functions beyond integrin-mediated attachment—including roles in cytoskeletal organization and cell polarity—it is difficult to exclude the possibility that the observed effects stem from broader cellular changes. The argument that the triangular mesh connectivity itself determines curvature would be more convincing if similar phenotypes were observed after perturbing other adhesion-related molecules (e.g., integrins, Kindlin, or ECM linkers such as Laminin). Demonstrating consistent outcomes via multiple genetic routes would provide stronger causal support for the proposed mechanism.

R3: We have followed the reviewer's advice and targeted other components of the basal adhesion machinery. Specifically, we have induced degradation of the basement membrane by expressing metalloprotease-2 (*GMR-Gal4>UAS-Mmp2*). The effects on basal patterning and on eye curvature are presented in Supplementary Fig. 7 and described on page 12. Mmp2 overexpression recapitulates the *talin-IR* phenotypes - effects on basal triangle meshwork and local curvature of adult eyes- strengthening our conclusion.

4. The fourth point is, in my view, the most critical issue in this study. The causal link between the triangular mesh and curvature remains somewhat indirect. The model provides a plausible mechanism, but the experimental data mainly show correlation rather than causation. Given that the central claim of the paper is that mesoscale geometry drives morphogenesis, a more direct test of this causal relationship would be essential to solidify the conclusion. While direct physical manipulation of the mesh may be technically difficult, indirect approaches could still be informative—for example, modulating hydrostatic pressure during pupal development, altering local tension optogenetically, or quantitatively comparing predicted and measured curvature gradients. If such experiments are not feasible at present, clearly stating that causality is inferred from modeling, and discussing this limitation explicitly, would make the argument more balanced and transparent. In my view, strengthening this causal analysis is the key to consolidating the scientific impact of this study.

R4: As the reviewer notes, the causal link between the pattern of triangle sizes and the specific variations in local curvature is firmly established in the computational setting, but much harder to establish in the biological system. A designed local variation in the size pattern leading to a change in local curvature predicted by the model would indeed be a very strong causal link. Therefore, we performed a functional experiment aimed at genetically reprogramming the pattern of triangles of the mesh. In it, we show how perturbation of the mesh locally (affecting just the dorsal region of the retina) perturbs the eye's local curvature. Reproducing this perturbation computationally also recapitulates the changes in local curvature. We believe these experiments, described on Results (page 10) and in a new Fig. 3, strengthen the causal link between basal triangular mesh patterning and control over local curvature. In any case, and to avoid overstatements, we state our argument in a more balanced and transparent fashion in Discussion (page 14) as: "However, the fact that our model is able to recreate the subtle local curvature of different compound eyes based solely on the pattern of triangle sizes, suggests that this pattern is the dominant cause of the local curvature modulations."

5. *A related point concerns the claim that "the triangular mesh is critical for the robustness of the 3D curvature." This assertion would be reinforced by showing that other mesoscale structures—such as apical cell patterning or the corneal lens layer—do not play comparable roles. Demonstrating that curvature remains intact when apical organization is perturbed but the basal mesh remains connected would provide compelling evidence that the basal structure specifically underlies robustness. If such experiments are impractical, the authors could still discuss why the basal mesh is most likely the principal determinant. This issue is conceptually linked to the previous point, as both address how the system ensures causal and structural specificity in curvature control.*

R5: Unfortunately, we cannot specifically affect apical patterning without affecting basal organization. This prevents us from testing a potential role for apical surface organization in curvature. However, we have analyzed quantitatively the apical hexagonal patterns of *talin-IR* retinas and found that their pattern is indistinguishable from controls (results shown in Supplementary Fig. 6). We take these results as further evidence that the basal triangular meshwork, rather than apical surface organization encodes local curvature. We have modified our Discussion, on page 15, to indicate that "The basal mesh seems to be better suited to encode retinal shape than the apical side. On the one hand, the basal surface is directly exposed to the pupal hydrostatic pressure. On the other, during pupal development, stress fibers build up along the elongated basal side of the secondary pigment cells, which form the triangle sides, which are known to integrate mechanically the retinal epithelium [35,36]."

Reviewer #1 (Remarks on code availability):

I checked the GitHub page, but the number of files was so large that I couldn't follow the entire contents.

R: We thank the reviewer for checking the GitHub repository. The repository includes a concise index file that outlines the structure of the codebase and that indicates the purpose of each script and pipeline. Each script is also individually documented. The relatively large number of files reflects the presence of multiple analysis and simulation pipelines developed in the course of the project. Given the scope of the computational work, the repository is intended to support the analyses presented in the manuscript, and individual scripts can be consulted as needed in relation to specific analysis steps.

Reviewer #2 (Remarks to the Author):

Overall opinion

In this study, Garrido-Garcia and colleagues describe an original morphogenesis model whereby prepattern of basal cell arrangement forming a network of connected structures encodes the 3D shape and curvature of an hemisphere upon inflation. This metamaterial provides a very relevant morphogenetic framework which applies very well to the morphogenesis of the compound eyes of Drosophila melanogaster and Drosophila mauritania, and can also explain the 3D shape upon perturbation of talin and the binding to ECM. The paper, relatively short, is mostly based on precise characterisation of the triangular pattern of the retina in the two species and upon perturbations of talin, and prediction of the emerging 3D curvature using argent based model as well as carefull quantitative comparison of the experimental and simulated 3D shape. The paper is original, well structured, easy to read, based on very clean (and non trivial) 3D characterisation and the results quite compelling. This is of course relatively correlative in many aspects, but it convincingly shows the relevance of the modelling framework. The results should be of wide interest in the field of morphogenesis and goes along the line of other recent results trying to dissect 3D morphogenesis in the framework of 2D deployable systems. I am overall very supportive for publication, but I do have few suggestions of few additions of experimental conditions that would help to strengthen the validity of the model as well as some clarification of the biological system and potential limitation/hypothesis in the model.

R: We thank the reviewer for their very positive evaluation of our work.

Major suggestions

1. *The model convincingly manages to recapitulate D melanogaster, D mauritania and talin depletion eye shape. However all these perturbations/genetic background relie on global effect that might be quite pleiotropic. Assuming that the local triangular pattern mostly has a local impact on curvature, it would be very relevant to include some clonal/local perturbations to see whether this leads to predictive local curvature changes. This might be difficult with clonal tools if it is not possible to image the triangular pattern/clones and the 3D eye shape in the same tissue (although this might be possible at late pupal stage ?), but it could be done at least using driver with predictive pattern affecting only a portion of the eye (e.g. : mirror-gal4).*

R1: We followed the reviewer's suggestion and used the *Iro-Gal4* (a dorsal-specific-GAL4 driver line) to drive *UAS-talin-IR* to the dorsal region of the developing retina. We found that the effects on basal retinal patterning were very mild, possibly because *Iro-*

Gal4 expression weakens posterior to the morphogenetic furrow, as the retinal tissue undergoes patterning. To circumvent this technical limitation, we sought to perturb the pattern of triangle sizes locally, by expressing *gigas RNAi* in the dorsal region of the retina (*Iro-G4>UAS-gigas-IR). gigas* is a negative regulator of cell growth. In these retinas, the dorsal region of the mesh has larger and, in cases, missing triangles (due to ommatidial fusion). This local perturbation (i) causes an alteration of the local curvature of the adult eye and (ii) when these mesh alterations are tested using our computational model, they also lead to a curvature distribution that match the local curvature of adult *gigas* eyes. These results are now included in a new Fig. 3. The alteration in curvature distribution is subtle, though, since the region affected is approximately a third of the eye surface and our metric compares the curvature distribution of the whole eye.

2. *So far the only perturbative condition provided is based on Talin depletion. The results are really convincing and the increase of 3D curvature tissue to tissue variability really compelling. Yet, talin may affect other parameters that could affect 3D tissue shape (e.g. : basal membrane tension, lateral tension, Rho activity, polarity…). To fully demonstrate that the triangular pattern plays an instructive role, it would be good to provide at least one alternative perturbative condition affecting the pattern and 3D curvature. For instance, would it be possible to affect the apex of triangles (the grommet) for instance by affecting locally IOC fate ?*

R2: In the revised manuscript, and following the reviewer's suggestion, we provide two new perturbations to complement our *talin-IR* experiments that support the instructive role of the patterned triangular mesh in the local modulation of eye curvature. One of them, which we have described in R1, was the local perturbation of the triangular mesh attenuating *gigas.* The other was a perturbation where the ECM is degraded by driving the expression of Mmp2, causing a total disruption of the basal mesh without major alterations on the apical retinal pattern. In these experiments both the triangular meshworks and the local curvature of the eye are affected, and the effects on the curvature distribution are recapitulated by our computational model. These results have been incorporated into the manuscript and shown as the new Fig. 3 (*gigas-IR*) and Supplementary Fig. 7 (Mmp2).

3*. The fact that triangle pattern is enough to recapitulate the 3D shape is not exclusive of an inverse relationship (3D shape imposing the triangle pattern). The image describing triangular pattern seems to emerge pretty late (around 40h APF) while the inflation/curving of the eye starts around 20h APF. To which extent the curving itself is influencing the triangular pattern? The author should clarify this and clearly document whether the basal pattern is fixed prior to curving/inflation.*

R3: We have clarified the sequence of events leading to formation of the mature retina (Fig. 1 and associated figure legend). We also have included a discussion (page 14) on this topic ("Our work indicates that the final local curvature of the adult eye, including its local variations, is acquired in two steps. (...)"). In summary, the retina is curved before the triangular meshwork is formed, likely by hydrostatic pressure building up within the pupa. However, this is a general curvature, not the finely tuned local curvature we measure in the adult eye. Therefore, as predicted from our computational modeling and measurements of 3D curvature, which captures local curvatures (Box), basal meshwork establishment is required for further local curvature adjustment - essential for animal ecology and physiology. The basal patterns emerges as programmed cell-death eliminate interommatidial cells (Brachmann and Cagan, 2003) and basement membrane patterning coordinates interommatidial cell positioning and basal geometry remodeling, which generates the triangular meshwork (Walther et al., 2024).

Regarding the influence of the curvature on the mesh pattern. This is a point raised also by another reviewer. To check whether the flat mounting of the retinas resulted in a distortion of the *in vivo* pattern (where the retina is curved by pressure), we imaged dorsal and ventral regions of live pupal retinas, intravitally and at the same stage that fixed retinas, which were mounted flat for confocal imaging. For our live confocal analysis, we could not image whole retinas since these are curved. So, we focused on dorsal and ventral regions, as these showed the largest difference in triangle size in fixed, flat-mounted retinas. Indeed, these regions show the same triangle area ratio in both fixed and live retinas, with larger dorsal triangles.
This new control is mentioned in the text and shown as Supplementary Fig. 2. These new results strongly indicate the triangle size pattern and arrangement in retinal development does not depend upon curvature.

4. If I am correct the metamaterial model assumes constancy of the material property of its unit (here the triangle). However I suspect that depending on tissue specific localisation, the quantity of ECM/actin may vary and the tensional state of triangles may not be totally homogeneous. Of course, this should be reflected in the shape/size of triangle that the authors can capture in their model and experimental data. Yet, I think it would be important to openly discuss this important reductionist hypothesis and its relevance to describe the system (at least in the discussion).

R4: This is indeed an interesting point. We acknowledge that the computational model assumes uniform material properties for the triangular elements and therefore represents a reductionist description of the system. Importantly, however, our results indicate that the spatial pattern of triangle size is sufficient to generate heterogeneous mechanical responses, even in the absence of explicit spatial heterogeneity in material properties. In the biological system, the graded size of the triangles observed along the dorsal–ventral axis may arise from differences in cell size and/or basal membrane tension. While the present model does not explicitly incorporate such sources of heterogeneity, it is nonetheless able to capture their mechanical consequences through geometry-dependent deformation patterns and to recapitulate key biological behaviors. We have clarified these points in the Discussion and highlight that elucidating the genetic mechanisms controlling triangle size represents an important direction for future work.

Thus, we discuss in page 14 that "Although the model assumes uniform material properties, it still is possible that these properties may vary across its basal surface *in vivo* due, for example, to local changes in the ECM amounts or composition, or to variations in contractile properties of the basal actomyosin cytoskeleton [35,36]. However, the fact that our model is able to recreate the subtle local curvature of different compound eyes based solely on the pattern of triangle sizes, suggests that this pattern is the dominant cause of the local curvature modulations.", and points that "A key question moving forward is which genetic mechanisms translate positional information into cell size regulation across the tissue during normal development."

Minor suggestions

1. *The concept presented in this article are not totally different from a recent study from Natalie Dye group (https://www.science.org/doi/10.1126/sciadv.adp0860) using the concept of programmable/deployable 2D system to from complex 3D structure (applied to the wing disc). I do acknowledge that the modelling framework and concepts are different (in their case using in plane gradient of strain), but both studies use the concept of programming 3D shape in a 2D pattern. It would be relevant to include this reference in the discussion.*

Rm1: We thank the reviewer for this suggestion. We are now referring to this work in the developing wing, drawing parallels and differences with our work in the developing retina.
This is now discussed as: "Recent work in the *Drosophila* wing has shown that it is the heterogeneity of cell behaviors (including changes in the cell's apical surface and cell neighbors) in the developing 2D wing epithelium that programs the 3D shape[37]. This contrasts with the mechanism we describe here for the eye, where a stereotypical supracellular pattern provides the basis for the fine-scale control of 3D curvature. In any case, these two cases illustrate that different organs may use distinct mechanisms for precise control of their shape, even within a single species."

2. *Figure Box d: Could the authors be more specific about the parameter calculated in the values on the arrows between conditions/simulation steps? The info can be found in methods, but it would help to quickly describe this in the legend.*

Rm2: We have included the explanation of the arrows and the numbers in the legend of the figure. We have amended the figure legend to give more specific details regarding the conditions of simulation. The numbers represent the values of the Gaussian metric between the two shapes that are pointed by the arrows. The most important thing to notice is that the smaller the metric, the more similar the shapes are.

Reviewer #2 (Remarks on code availability):

I didn't review the code and cannot comment this section.

Reviewer #3 (Remarks to the Author):

Overall opinion
This paper by Garrido-Garcia et al. investigates how the basal cellular pattern of the Drosophila pupal retinal epithelium influences regional anisotropies in adult retinal curvature. Specifically the paper considers how isotropic pressure, exerted on a non-uniform triangular "mesh", might induce a 3-D morphology with an anisotropic distribution of local curvatures. They implement a mathematical framework that allows them to apply pressure to a 2-D mesh with specific geometry and then examine the resulting 3-D shapes using a Gaussian curvature metric. Using a 2-D mesh that matches the network of secondary pigment cell (PC) basal contacts in a wildtype pupal retina, they show that pressure-induced inflation of this flat surface results in a half-dome structure that recapitulates the regional curvature anisotropies observed in adult fly eyes. Disruption of basal pattern via RNAi knockdown of the integrin associated protein Talin results in regional curvature defects in adult eyes and in matching simulations. They conclude that tissue-scale basal pattern in the pupal retina critically informs regional curvature anisotropies in the adult eye. Overall, the question of how specific cellular morphologies channel forces from the growth environment to produce functional organ morphologies is of broad general interest. Further, the modeling framework is a clever way to address tissue properties and relationships that are hard to measure and manipulate in vivo. Thus, the work has the potential to be of interest outside of the fly eye field.

However, I have concerns about the approach and some of the conclusions, and so do not recommend publication of the manuscript in its current form. Of greatest concern, the main premise of the modeling experiments does not match the known in vivo developmental progression of the retina. Second, the limited in vivo validation of

concepts learned from the computational modeling contributes to a general tendency to over-interpret experimental results and to overstate what should be more speculative conclusions.

A third important point is that throughout the text, the general term “curvature” is used to mean both the organ-scale curvature of the eye and the finer-scale anisotropies in local curvature – using language that clarifies more precisely these different concepts and scales and that distinguishes between curvature acquisition, refinement and maintenance, is essential to improving the accuracy with which the data are represented, interpreted and discussed.

R: We are glad to know that the reviewer considers the question that we address of general interest and our computational model a clever way to address it. We have dealt in detail with the reviewer's main concerns (representativeness of the modelling and potential over-interpretation of the results) in the following sections. In addition, we have amended the text to clarify that the model does not attempt to recapitulate every biological step, but to demonstrate that a 2D mesh of triangles can refine local curvature of a generically convex surface. We also concur with the reviewer that our nomenclature has been ambiguous. To address the reviewer’s comment, we have adopted a consistent term to designate the small-scale curvature variations or heterogeneities, “local curvature”. We have also clarified the timeline of retinal morphogenesis.

Major suggestions:

1. The mathematical modeling simulates the acquisition of 3-D curvature by applying pressure to an initially flat 2-D surface to induce out of plane bending. The input is a patterned 2-D surface with a triangular mesh derived from the basal pattern of secondary PC contacts typical of a 42h APF retina. However, recent work from the lab of one of the corresponding authors of this study shows that by 42h APF the tissue has already acquired its organ-scale 3-D curvature (Lancaster et al. doi: 10.1016/j.cub.2025.05.048). Indeed the Garrido-Garcia et al. manuscript references this prior paper and states “the thin pupal retinal epithelium becomes curved around 20 hours after pupa formation (hAPF), under the action of hydrostatic pressure”. Compounding the model vs. biology discrepancy, another previous paper from the same group (Walther et al. doi: 10.1371/journal.pbio.3002783) shows that at ~20-24h APF, stereotyped basal pattern has not yet been established and that PC basal contacts are highly disordered (these observations corroborate those reported in many earlier papers, starting with Longley & Ready 1995 (doi: 10.1006/dbio.1995.1292)). Therefore, the central premise of the modeling experiments and of the paper overall, that pressure acts on a flat 2-D surface with a specific, stereotyped pattern does not match the developmental progression of retinal basal patterning and curvature acquisition seen in vivo. This discrepancy calls into question the validity of the study as currently framed.
It seems formally possible that basal pattern (which is acquired after tissue-scale curvature establishment, according to the timeline presented in Fig 1E and previous work from multiple labs that have characterized basal pattern formation) refines pre-established retinal curvature and is responsible for the local curvature anisotropies in adult eyes as proposed. By performing similar tissue-scale Gaussian curvature measurements on pupal retinas both before and after basal pattern refines (24h vs 42h APF) the authors could test this. If basal patterning refines tissue curvature, the prediction is that curved retinas at 24h APF would display gaussian curvature metrics that differ from those of adult eyes, while 42h APF retinas would not. The computational model would also need significant revision to incorporate the initially disordered basal pattern (known to be present in the flat tissue) and the subsequent acquisition of the basal triangular mesh in an already curved surface. Such experiments would allow the

authors to define more clearly the principal question and conclusion of their study, as the resultant data would provide evidence that basal pattern is required for regional curvature refinement but does not influence organ-scale curvature acquisition. This interpretation would also be consistent with the result that Talin RNAi retinas (which have perturbed basal pattern) produce local curvature anisotropy defects, but have an intact tissue-scale "half-dome" structure.
Another formal possibility that might reconcile the current disconnect between model and biology is that the influence of internal hydrostatic pressure might only become relevant after the basal network pattern has refined. However to my knowledge this is not known and so can't be assumed, and testing it experimentally seems challenging.

R1: The reviewer's issues make reference to three important aspects.

First, the reviewer is right in that retina inflation ("general curvature") starts before the basal patterned mesh is established, while our computational simulations start inflation with the pattern already established 2D (Fig. 2c). Then, it progressively curves after each computational iteration. Therefore, from the flat first iteration to the final iteration (sWT) there are hundreds of intermediate steps. After the reviewer comment, we have tested if the model can reproduce the two-steps biological process. Although the final curvature distribution of sWT and sRub (simulating the patterned triangular mesh and a homogeneous elastic material, respectively) end up being very different, we reasoned that if both meshes passed through an intermediate iteration where their curvature metrics were very similar, then the local curvature differences might arise later. Indeed, this is the case (see "Reviewer Table 1" below, where 6 pairs of sWT and sRub were simulated). We found a very similar inflation (difference between the ratio depth/major axis < 2%) for each pair of sWT and sRub for which the curvature distributions of the two surfaces were very similar (Gaussian metric sWT/sRub < 0,09). The column "Matching iteration sWT/Total iteration sWT" indicates the specific iteration of the sWT simulation at which inflation and curvature metrics match. Therefore, this analysis indicates the patterned mesh becomes relevant from mid inflation onwards, which is compatible with the biological timeline. In addition, this control validates the computational analyses presented in the paper, as the same final curvature distribution will be reached if the pattern is present from the onset of inflation or midway.

Simulation Name	Difference of Ratio depth to major axis between sWT/sRub	Gaussian metric sWT/sRub	Matching iteration sWT/Total iteration sWT
**sWT/sRub 1 Female**	0.014	0.074	182/298
**sWT/sRub 2 Female**	0.011	0.036	120/211
**sWT/sRub 3 Female**	0.008	0.054	143/194
**sWT/sRub 1 Male**	0.0003	0.087	198/261
**sWT/sRub 2 Male**	0.011	0.065	132/287
**sWT/sRub 3 Male**	0.003	0.089	236/458

Reviewer Table 1. sWT and sRub pass through an intermediate inflation iteration where their curvature metric is nearly identical. Global shape similarity, quantified by the Ratio depth to major axis and the Gaussian metric values, indicates that for each retina there is a pair of iterations in which the sRUB model matches the sWT model (Matching iteration sWT). The last column shows the iteration in which the matching occurs

compared to the total of iterations for each sWT. This is the point that would correspond to the developmental stage at which the general curvature has been established.

Second, the issue of the distinction between general curvature on the one hand, and small-scale curvature variations on the other. Our paper deals with the following question: what is the mechanism by which small-scale curvature variations are programmed in the developing retina. We set out to address this question in the opening sentence of the abstract ("The panoramic vision of the convex compound eyes, common to insects and crustaceans, relies on micrometer-scale curvature variations. These variations generate specialized visual zones adapted to specific tasks, including detecting prey, mates, or predators. However, how such fine-scale curvature is encoded during development remains unknown.") and the first paragraph of the Introduction ("Importantly, curvature can vary across the eye, and these small-scale curvature anisotropies (what we will call heretofore "local curvature"), which make the eye deviate from a uniform dome, often differ between species[1].(...)). Therefore, the mechanism that we describe does not deal with the process of general curvature -which we have assumed, based on prior work, to be driven by hydrostatic pressure. In any case, it is clear that our previous version did not make this distinction clear. Now we have made amendments through the text to make sure that the reader will know our paper addresses local curvature variation of the eye, which is critical for visual performance. In order to be consistent, these local curvature variations have been named as "local curvature".

The **third** aspect refers to the difference in curvature at different developmental times. General curvature of the retina increases steadily from 20 to 42hAPF, as has been reported by Lancaster et al., 2025. The reviewer rightfully notes that, if our mechanism were correct, a change in *local curvature* should be detected between 20 and 42 hAPF, as the patterned triangular mesh appears during this period. We have carried out an analysis to test specifically this point, following the reviewer's suggestions. We aimed at quantifying the curvature distribution in live pupal retinas at two time points: at 21 hAPF, when the retina is already curved, and at 44hAPF when in addition to this general curvature, a curvature modulation that should approximate the curvature distribution of the adult eye should be detectable. For this, we used lightsheet microscopy to obtain 3D volumetric reconstructions of live retinas with minimal disturbance. However, we found 3D penetration across the whole eye was incomplete and whole retinal imaging was not feasible. To circumvent this technical limitation, we analyzed 2D optical sections from the pupal retinas imaged with lightsheet microscopy -as this technique allowed us to reach and image the central section of the retina. For this analysis we have adapted our gaussian curvature metric to 2D curved silhouettes (Mean Apical Curvature, MAC. We include below a detailed explanation of how this metric is computed). We have also generated this metric for 2D silhouettes from 3D adult eyes to make the comparisons between 21hAPF, 44hAPF and adult shapes. We were aware that by reducing one dimension the curvature information might also be reduced. But we hoped that still some curvature information remained. The results are plotted in "Reviewer Fig. 1". Indeed, MAC values are significantly lower at 21 than at 44 hAPF. Remarkably, and in very good agreement with our model that basal surface patterning encodes for local curvature, we found the curvature measurements at 44h APF (but not at 21 hAPF) are very similar to the adult WT analyzed with the same methodology (2D MAC distributions did not differ statistically). This supports the notion that curvature refinement occurs between 21 and 44 hAPF, the time period when the basal pattern is established.

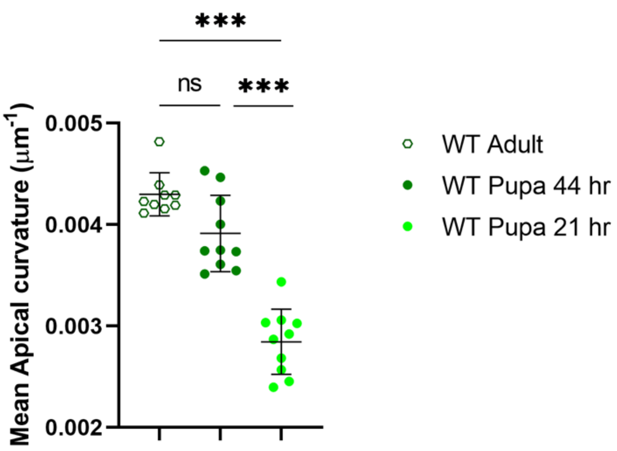

Reviewer Fig. 1. 2D Mean Apical curvature (MAC). From light sheet confocal microscopy stacks, the optical section corresponding to the maximal extent of the apical retinal surface was identified and manually segmented to obtain the anterior–posterior apical contour and the mean apical curvature (MAC) was extracted for each retina. MAC values are significantly lower at 21 hAPF than at 44 hAPF, with no statistically significant differences between WT and 44hAPF MAC distributions. These results indicate that curvature refinement occurs between 21 and 44 hAPF.

2. *The authors state two ideas central to their proposed model as accepted facts. In my opinion, there is insufficient experimental validation to make these claims so strongly. First, the authors state that pressure induces curvature, and cite Lancaster et al., 2025. To ask if positive pressure within the developing pupa influences retinal curvature, Lancaster et al. punctured the pupal case distal to the head capsule, and noted tissue-scale defects in retinal curvature. There is not sufficient methodological detail provided to fully understand the experimental protocol or the developmental stage at which the puncture was performed, but visually it appears it was made at a stage of development when curvature has already been acquired; if so, then puncturing the pupal case perturbs established curvature. Therefore from this experiment alone, the only non-speculative conclusion is that pressure is required for curvature maintenance. It is interesting and appropriate to speculate the pressure might induce curvature, and might be required to refine local curvature while maintaining tissue scale form. Since measuring dynamic changes in developing pupae is technically challenging, the approach of exploring the hypothesis that pressure induces curvature with a computational model is appropriate, but the authors need to adjust the language throughout the manuscript to acknowledge the speculative nature of proposed models and to acknowledge that future in vivo experiments will be needed to validate the predicted biology.*

R2: Classic works propose that an increase in hydrostatic pressure in the early pupa of Cyclorraphan flies, which includes *Drosophila*, generated by abdominal muscle contraction, is responsible for head eversion, a major morphogenetic event that not only results in the unfolding of the head from the notum, but in the convex curvature of the head structures, including the eye (Wall, 1914; Robertson, 1936; Bodenstein, 1950). This would place hydrostatic pressure as a primary driver of curvature. Further, the puncturing experiments performed in Lancaster et al., 2025 were made at 20h APF, which is consistent with this pressure being required for curvature. If pressure were not the primary force acting, curvature would need to be induced by yet another force. At this stage we favor the simplest model, compatible with the evidence available to us. We

have amended the manuscript in line with this reviewer comment, to ensure we do not over interpret our results.

In the first paragraphs, we introduce hydrostatic pressure as the most likely force driving curvature (Page 2) as "This transformation likely starts with the building up of hydrostatic pressure within the pupa due to the contraction of the abdominal muscles. This pressure would provoke the eversion of the head, including the developing retina, and the acquisition of a general convex curvature at around 20 hours after puparium formation (hAPF) [7-10]. Puncturing experiments performed by Lancaster and coworkers [10] show that a reduction of internal pressure as early as 20 hAPF results in loss of retinal curvature, supporting this idea. Retinal curvature keeps increasing steadily until 45-50 hAPF. From this time point, the retina will further increase its curvature very little [10]. After metamorphosis, the convex adult *Drosophila* eye shows a non-homogeneous curvature, as expected for the eye having local visual specializations (Fig. 1a,b). How the small-scale variations of the eye curvature are encoded in the fabric of the retinal tissue is not known."

Second, in the introduction the authors describe the crystalline lens as a "hard polymer" and state that its secretion locks the organ scale curvature in place and prevents any further morphological changes. While this seems plausible, neither experimental validation nor references are provided. To my knowledge there is only speculation in the literature on these points. Normally I would recommend simply removing this statement, but the idea is important to the proposed model. Specifically the authors propose that the initial triangular pattern of secondary PC basal feet establishes early in development the curvature anisotropies observed later in the adult, and that the crystalline lens locks in these early regional differences in curvature throughout subsequent phases of development when the IOC basal pattern changes substantially. At a minimum, the authors need to confirm in vivo that the curvature anisotropies seen in the adult eye are present at a stage in pupal retinal development where basal pattern of the retina is established, but the vitreous lens has not been secreted (~40h APF). In other words, they need to define exactly when the fine-scale curvature differences seen in the adult are actually established in the developing retina.

R2b: Our computational model and genetic experiments indicate that the basal triangular meshwork that arises as the interommatidial cells get into place and shape, encodes for curvature anisotropies. We do not know what mechanism "keeps these anisotropies in place" once they are established. It is possible that, as we mentioned, the cuticular lens does this. It is equally possible that further basal regulation is responsible -for example, basement membrane remodeling or other mechanisms.

To address the reviewer's comment, we now offer a short discussion on this topic (page 15): "The basal mesh seems to be better suited to encode retinal shape than the apical side. On the one hand, the basal surface is directly exposed to the pupal hydrostatic pressure. On the other, during pupal development, stress fibers build up along the elongated basal side of the secondary pigment cells, which form the triangle sides, which are known to integrate mechanically the retinal epithelium [35,36].". However, we would like to stress that here we report a mechanism to establish local 3D curvature anisotropy. We are not addressing what maintains these anisotropies in the "homoeostatic state", which we feel is very interesting, but outside the scope of our present study. In addition, as we have described above, our new analysis of *in vivo* curvature at two time points (before and after the establishment of the basal triangular pattern) shows how the curvature distribution of the retinal epithelium changes during development to approximate that of the adult eye (Reviewer Fig. 1).

3. Related to both concerns discussed above in points 1 and 2, there is a tendency throughout the manuscript to make overly general statements and to over-interpret experimental results without the necessary in vivo validation. Therefore, claims made in this paper need to be tempered to more accurately reflect conclusions appropriate to the data presented. The discrepancy in the timing of tissue-scale curvature acquisition and basal pattern formation underlies the most serious examples. For example, the discussion states that this paper uncovers a "novel strategy to program a 3D shape in vivo: uniform hydrostatic pressure acts on a patterned non-homogeneous tissue." I also found it inappropriate to claim discovery of a "new level of organization" at the basal surface. The pattern of 2oPC feet has long been noted; what is new and exciting in this study is the idea of considering it as a 2-D patterned mesh, or metamaterial, that templates 3-D tissue properties.

R3: We have amended our manuscript to ensure no section comes across as over-interpretation and making it clear when we speculate in our revised discussion. In particular, we have changed the two sentences that the reviewer mentions above. We restrict our main claim to the discovery that the retina can work as a metamaterial using a 2D patterned mesh to encode for local curvature variation in a reproducible manner. Thus, we summarize our results (page 12) as "Altogether, our results identify the basal multicellular triangular mesh of the pupal retina, with its pattern of triangle sizes, as a major mechanism encoding the fine-scale local curvature variations of the compound eye and its robustness.".

Minor suggestions

1. The authors argue that in order for the basal pattern to be considered a meta-material that regulates retinal curvature, it must be stereotyped. Presumably the authors mean that this pattern should be present with little variability across a population of retinas? In the supplemental materials, they show retinas from one male and one female fly, and the two triangular meshes do appear similar. However, it seems important to account for population scale variability, and so triangular mesh structures should be segmented from a larger population of animals and a statistical analysis of the spatial variability should be calculated. This average mesh structure should then be used in the simulations.

Rm1: We acknowledge the description of this part of our method was not clear: We have segmented the triangular mesh of *all* the samples that we have analyzed to perform the individual simulations and obtain individual results that can be later compared with the adult eyes. As the reviewer mentions, it is the population level distributions which are most informative, as they reflect, beyond the average, the real variability of patterns and local curvatures. To summarize: we have segmented and quantified all pupal retinas (6 WT; 4 Mau; 6 Gigas; 6 Talin; 6 Mmp2) and generated an equivalent set of computationally-generated "eyes" so that proper statistical contrasts could be applied (Supplementary Data 5).

2. The authors segment a planar (2-D) triangular mesh pattern from 42hr APF retinas. As stated in this paper and by Lancaster et al. 2025, these retinas have already acquired a stable tissue-scale curvature. Additional methodological detail is needed to understand how they segmented the planar mesh from this curved surface. If 42hr APF retinas were flattened to image basal gromets and segment the triangular mesh structure, control experiments demonstrating that flattening does not perturb or distort the PC shapes need to be included. As spatial features of the pattern are critical for the curvature that the

computational model generates (although not actually present in vivo when the curvature is acquired) clarification of this point is important.

Rm2: We thank the reviewer for raising this point. The retinas from which we extract the 2D pattern of triangle sizes are mounted flat. Although this pattern is sufficient to explain the local curvature of adult eye surfaces, it was an important control to verify that the dorsal ventral pattern of triangle size we imaged in flat retinas was also present in live ones of the same stage (42hAPF). In the latter, as the tissue is curved, we cannot image the whole retina, but could sample smaller regions instead. We decided to focus on the dorsal and ventral areas of the retina, where the triangle size differs the most, and compared the size ratio in both fixed, flat mounted retinas and live retinas. Our quantitative analysis shows that indeed the size ratio is the same in both cases, indicating that mounting does not distort the pattern. We provide this new control as Supplementary Fig. 2.

3. The authors use RNAi-mediated knockdown of Talin to perturb basal pattern, and find regional curvature anisotropy defects in both the computational model and in adult retinas. Talin provides a mechanical link between cytoskeletal structures in the cell interior and integrin complexes that interact with extracellular matrix (ECM) structures. It has been demonstrated in the developing fly retina that cell-ECM interactions form a network with contractile properties at the basal surface of the retinal epithelium (Ready & Chang, 2023, doi: 10.1016/j.cub.2023.04.066). Thus Talin knockdown might also disrupt basal cell mechanics (in addition to basal pattern). Given this possibility, the conclusion that basal patterning defects induced by Talin knockdown are the sole driver of the resultant curvature anisotropy defects is too strong a statement.

Rm3: We agree with the reviewer in that Talin RNAi might alter tissue mechanics beyond having a role in allowing the formation of the 2PC triangles. However, this should not be a problem with our hypothesis. When Talin is present, the mechanics of the cells (which will be important for the general mechanical properties of the tissue) are modified non-homogeneously, as the size of the triangles also have a non-homogeneous distribution. This fact would be sufficient to modulate the curvature distribution of the tissue. A tissue without a patterned mesh (like in *talin-IR*) should behave like a homogeneous material (similar to rubber), to some extent independently of its detailed mechanical properties; provided that it is sufficiently elastic to endure curvature by inflation. The similar curvature distribution between *talin-IR* eyes and the curved surfaces derived from our model simulating a rubber-like material is compatible with *talin-IR* retinas being a uniform material, although we cannot tell whether its properties would differ if the cells were expressing Talin.

In any case, to address this comment, we carried out an additional perturbation in which the ECM and the basal attachment were not primarily disturbed. In this experiment, by driving a *gigas-RNAi* to the dorsal third of the retina, we changed the size and pattern of the triangles. Even this relatively mild change in pattern resulted in local curvature changes we could both measure in the adult animal and simulate using the 2D mesh we extracted from *gigas-IR* retinas. These results are now presented in a new Fig. 3. While it is possible cell size changes affect cell-ECM interactions indirectly, we think these new experiments in which it is the pattern of triangles, not the mechanical attachments to the ECM, that is perturbed, are consistent with our main conclusion that the 2D basal pattern of triangle size is the major factor in setting the final, precise curvature of the retina. In any case, and in full agreement with the reviewer, we cannot claim that this is the only mechanism at play. We have toned down the text to be less taxative (page 12): "Altogether, our results identify the basal multicellular triangular mesh of the pupal retina,

with its pattern of triangle sizes, as a major mechanism encoding the fine-scale local curvature variations of the compound eye and its robustness."

4. The use of the Gaussian curvature metric is prominent throughout the paper, but the details of how it captures the regional differences in tissue curvature that this study focuses on are not sufficiently clear. To facilitate broader readability, this method should be explained in greater detail, focusing on how this mathematical concept is employed and how it captures regional curvature features of the retina. Some statistical comparison of model output to in vivo measurement should be included.

Rm4: We anticipated introducing a new method to measure 3D curvature -something that is a complex matter- would require us to offer a detailed account of it. We therefore provided a dedicated Box to specifically walk the reader step-by-step through this procedure. We have revised the legend of this Box to make it as clear as possible. In addition, the mathematical formulation of the Gaussian metric is presented in the Methods section (Shape similarity metric, Gaussian metric). Regarding the statistical comparisons, several figures in the manuscript, including Fig. 1-3, account for this when analyzing the similarity between the resulting models and in vivo adult data. In particular, former Fig. 3 (now Fig. 4) explains with an example how the metric is computed (Fig. 4e, f).

5. It looks to me like apical geometry in the Talin knockdown is altered, contrary to what is stated. Specifically the ommatidia appear more diamond shaped rather than hexagonal shaped as they are in the wildtype image in 1G. Perhaps the selected Talin RNAi image in 3B is not representative of the result? Statistical comparisons of apical geometry should be performed, and the selected image (and statement in the text) should reflect the results.

Rm5: Following the reviewer's advice, we quantified the organization of the apical surface of *talin-IR* retina, compared to wild type. This new analysis shows that apical surface organization is minimally affected when we disrupt integrin adhesion signal *talin-IR*. These data are presented in Supplementary Fig. 6. In the revised text (page 11) we now state "The quantification of the hexagonal pattern on the apical surface of these retinas revealed the apical patterning remained largely unaffected (Fig. 4b, Supplementary Fig. 6; and [12,13,28])".

6. Justification (or mention of potential caveats) for using GMR-Gal4 to drives Talin RNAi in all retinal cells when the focus is on the secondary PCs should be added.

Rm6: We have chosen GMR-Gal4 as a driver since when overexpressing *talin-IR* the basal pattern was abolished without significantly altering other features of the ommatidial cells, such as their apical packing (Fig. 4b, c and Supplementary Fig. 6).
We have included a clarification on the Results section page 11: "*GMR-Gal4* is expressed in all retinal cells. In *talin-IR* retinas, the detachment of basal cells from the extracellular matrix causes the disconnection of the triangle mesh (Fig. 4a and Supplementary Fig. 6)."

RESPONSE TO REVIEWERS

Reviewer #1 (Remarks to the Author):

1. The revised manuscript does provide more technical information about the FEM model, including clarification of the t/h ratio and the use of thin-shell theory. However, my main concern from the previous round remains. The model is built on the assumption that the basal meshwork is the principal structure supporting tissue mechanics and bearing hydrostatic load, but this point has still not been demonstrated directly. At present, the manuscript does not provide direct experimental evidence that this multicellular basal mesh—rather than other components, such as the broader ECM or the cell bodies—is the main load-bearing structure in vivo. The simulations are useful and internally consistent, and I find them valuable as a theoretical framework. Still, without some physical measurement of the tissue mechanics—for example by AFM, micromanipulation, or laser ablation—it is difficult to know whether the assumed material properties of these triangles, and their proposed mechanical role, correspond to the actual biological system. At minimum, I think the manuscript should state this more explicitly and present the structural assumptions of the model as hypotheses that still require experimental validation.

Answer: We have made sure that we have not overstated our findings. Specifically, and to acknowledge a valid point made by the reviewer (that our model assumes constant mechanical properties of the cellular triangles throughout the retina) we have added the sentence:

Page 15: “Future experiments to physically measure the mechanical properties of the tissue will be necessary to challenge the structural assumptions of our simulations.”

2. The gigas RNAi experiments are also a welcome addition, since they address one of the key issues raised in the previous round. They provide an important functional test. Even so, I do not think they yet validate the full biological mechanism proposed by the authors. As presented, the simulations show that the geometric pattern, even under uniform material properties, is sufficient to reproduce the observed curvature. That is an important result. But it is not the same as showing that the pattern is the necessary cause, or even the dominant cause, in the living tissue. The authors themselves acknowledge that other factors—such as spatial differences in contractility or ECM composition—may also contribute in vivo. For that reason, I would encourage more restrained language here. The pattern may well act as a major guiding factor, or as a geometric template that works together with other mechanical properties, but the current data do not justify presenting it as the dominant cause.

Answer: Following with the general suggestion above, we have used language that indicates that our mechanism is likely the major guiding factor in controlling local curvature (something that seems to have convinced the two other reviewers), but by no means excludes the contribution of some other factors. We name a few, related to spatial heterogeneities in the extracellular matrix.

Page 10: “Globally, all these results support the notion that the pattern of triangle sizes is, at least, a major guiding factor of the fine-scale local curvature of the eye.”

Page 14: “Although the model assumes uniform material properties, it is still possible that these properties vary across its basal surface *in vivo* due, for example, to local changes in the ECM composition or biophysical properties, as well as to variations in contractile properties of the basal actomyosin cytoskeleton”

3. I also still see a mismatch between the revised Discussion and the Abstract. In response to my previous Major Suggestion 5, the authors appropriately softened the interpretation in the Discussion and now describe the basal mesh as a “major mechanism” rather than the sole determinant. However, the Abstract continues to state that the findings “identify a previously unknown mechanism of morphogenesis control whereby supracellular 2D patterning endows a tissue with genetically programmable metamaterial properties to encode fine-scale 3D curvature with high precision.” This echoes Reviewer 3’s concern about overly deterministic language. In my view, this wording remains too strong. Saying that the patterning itself “endows” the tissue

with these properties implies a degree of exclusivity and determinism that is not fully supported by the data, especially given the authors' own acknowledgment that the mesh is unlikely to be the only relevant factor. I therefore ask that the Abstract be revised so that it matches the more measured tone now used in the Discussion and better reflects the multilayered nature of morphogenesis.

Answer: The reviewer is right in that the language used in the abstract was more assertive. We have changed it to use the same tone as in the rest of the paper.

Reviewer #2 (Remarks to the Author):

The authors have adressed all my concernes and significantly improved the manuscript, notably by providing novel perturbative conditions and using local perturbation. The thorough discussion about the timing of inflaction, basal pattern establishment and refinment of the curvature was also very information. I am now fully supportive for publication and would like to congratulate the authors for this very nice work.

Answer: We thank the reviewer for the positive comments about our work.

Reviewer #3 (Remarks to the Author):

The authors have done a very thorough job addressing reviewer comments. The new data, controls and text that have been added have greatly improved the rigor and thoughtfulness of manuscript. I have no further concerns and recommend publication.

Answer: We thank the reviewer for the positive comments about our work.
